# Lived Experiences of Nurse Migration to High‐Income Countries: A Qualitative Inquiry

**DOI:** 10.1155/jonm/1582171

**Published:** 2026-06-25

**Authors:** Ayise Karadag, Memnun Seven, Aleyna Uçanbelen, Nichola Ashby

**Affiliations:** ^1^ School of Nursing, Koç University, Istanbul, Türkiye, ku.edu.tr; ^2^ Elaine Marieb College of Nursing, University of Massachusetts Amherst, Amherst, Massachusetts, USA, umass.edu; ^3^ Graduate School of Health Sciences, Koç University, Istanbul, Türkiye, ku.edu.tr; ^4^ Horizon Health Consultancy, Leicester, England, UK

**Keywords:** nurse migration, nursing workforce, professional integration, qualitative study

## Abstract

**Background:**

Nurse migration is increasing worldwide, raising critical challenges for workforce sustainability, regulation, and integration in both home and destination countries.

**Aims:**

This study explores the experiences of Turkish nurses who migrated to the United States of America, the United Kingdom, and Germany, focusing on motivations, enabling factors, and challenges during immigration and integration.

**Methods:**

A qualitative descriptive design was employed, and 23 Turkish nurses were recruited through purposive sampling. Data were collected via semistructured interviews conducted on secure video platforms.

**Results:**

Content analysis revealed five themes. *The decision to migrate* reflected motivations of low pay, workplace violence, limited professional opportunities, and lack of respect, which collectively diminished nurses’ professional self‐worth in Türkiye. *The immigration process* highlighted barriers in language, licensing exams, credentialing, and visas. *Life after immigration* involved facing discrimination, adapting to a new culture, and pursuing professional growth. *Reflections and outlook* described overall satisfaction with the decision and future personal and professional career plans. *Recommendations* underscored the need for streamlined credentialing and stronger integration supports in destination countries, alongside improved workplace conditions, career opportunities, and respect for nurses in Türkiye.

**Conclusion:**

This study underscores the multifaceted drivers of nurse migration and calls for reforms to strengthen retention in Türkiye, while urging destination countries to adopt multidimensional support for integration to ensure a stable global nursing workforce.

**Implications for Nursing Management:**

Nurse managers in source countries must lead systemic reforms addressing the conditions that diminish professional self‐worth—unsafe workloads, low pay, workplace violence, and lack of professional respect—to meaningfully reduce migration intent. In destination countries, nurse managers are central to designing structured onboarding, mentorship, and culturally responsive integration pathways that support the adaptation and long‐term retention of internationally educated nurses.

## 1. Introduction

Nurses are essential to global health systems, yet major workforce imbalances persist. Despite growth from 27.9 million in 2018 to 29.8 million in 2023, the world still faces a 5.8‐million shortage, projected to decline to 4.1 million by 2030 [1]. Increased nurse migration from low‐ to high‐income countries provides workforce relief but undermines the stability of source health systems [2–4], thereby widening global inequities in nurse distribution [1]. Nurse migration poses distinct challenges for nurse leaders in both source and destination countries. In source countries, mass emigration depletes teams and forces nurse leaders into cycles of recruitment and reorientation [2]. In destination countries, nurse leaders are responsible for integrating internationally educated nurses into unfamiliar systems, and when this fails, early attrition compounds the very shortages recruitment aimed to solve [4, 5].

Leading destinations such as the United States, the United Kingdom, and Germany use streamlined licensure pathways and active recruitment to address nursing shortages [6]. While the United States and United Kingdom attract nurses with competitive salaries, Germany’s rapid growth in the number of foreign‐trained nurses in its workforce is helping stabilize ongoing shortages [7]. Although lower middle‐income countries such as the Philippines, Kenya, Nigeria, and Ghana are among the top source countries [6], emigration from Türkiye has intensified, with 76.3% of nurses expressing intentions to migrate, primarily due to low wages and limited professional opportunities, challenges heightened in the post–COVID‐19 period [8]. As nurse emigration from upper middle‐income countries grows rapidly, with economic instability, political uncertainty, and professional devaluation converging to erode nurses’ dignity and self‐worth, Turkish nurses offer a timely and important case for understanding how these forces shape migration decisions and the reconstruction of professional identity across multiple destination contexts.

Migration motivation can be understood through both classic push–pull forces [9] and contemporary labor mobility dynamics, where structural, professional, and personal factors interact to shape decisions [10]. However, migration rarely occurs without substantial push factors in the country of origin [11], operating across macro‐, meso‐, and microlevels where structural, social, and individual motivations intersect [10]. Despite extensive research on nurse immigration, the literature often separates structural drivers (e.g., economic/political forces) from nurses’ individual lived experiences [12–14]. A few studies have compared nurses’ experiences across multiple destination countries [5, 15], and limited attention has been given to how systemic and professional barriers jointly shape migration decisions and integration [4, 14, 16]. In particular, professional identity, dignity, and self‐worth, which are central to understanding why nurses leave and how they rebuild their professional selves abroad, remain underexplored [5, 17]. Consequently, an important gap remains in understanding how multidimensional factors shape nurses’ decision‐making, adaptation, and professional identity reconstruction [5, 18], a gap that is both a research priority and a leadership imperative, particularly among nurses from Türkiye migrating to the United States, the United Kingdom, and Germany.

### 1.1. Aim of the Study

This study aims to understand the lived experiences of Turkish migrant nurses across multiple destinations. The study seeks to explore immigration processes, struggles with systemic barriers, and subsequent integration into new professional careers among Turkish nurses who have migrated to the United States, the United Kingdom, and Germany.

## 2. Methods

This study employed a qualitative descriptive design to explore migration experiences in depth, including the immigration process and the meanings individuals assign to it. This approach is well suited to generating rich, naturalistic descriptions of participants’ interpretations, including identity transitions, motivations, and challenges inherent to migration [19, 20].

### 2.1. Participants

The study included 23 Turkish nurses who had migrated to the United States (*n* = 10), the United Kingdom (*n* = 6), and Germany (*n* = 7). These three countries were selected because they represent major global destinations for nurses, with active recruitment programs, distinct health systems, and high demand for the workforce [6, 7]. Notably, they differ substantially in nursing education requirements, regulatory structures, and licensure pathways [21–23], which contextualizes the varied integration experiences (Supporting Table 1).

Using purposive sampling, the eligibility criteria were being a nurse who immigrated from Türkiye to the United States, the United Kingdom, or Germany, having a nursing degree in Türkiye, and being employed as a nurse in the destination country. Nurses who immigrated for short‐term purposes (e.g., education) were excluded.

### 2.2. Data Collection

Data were collected through semistructured interviews and an online survey (Qualtrics) that included sociodemographics, professional background, and immigration details.

Interviews were conducted in Turkish via secure video conferencing (45–90 min) by the bilingual primary researcher (AK). Transcripts were generated using Zoom’s automated transcription feature and then reviewed and verified in Turkish by a research assistant (AU), a native Turkish speaker with good English proficiency, who also translated them into English. Later, English translations were reviewed and verified by a bilingual faculty member (MS) and an immigrant nurse, both native Turkish speakers with extensive professional experience in the United States. This dual‐review process ensured linguistic accuracy and preserved the cultural and contextual meaning of participants’ accounts, strengthening the trustworthiness of the translated data.

Interviews began with warm‐up questions and then moved on to migration, integration, challenges, and expectations. The semistructured interview questions are provided in the Supporting Table 2 (Supporting/Table 2). Data collection continued until saturation was reached. Saturation was assessed independently within each country group (United States, United Kingdom, and Germany) by monitoring for redundancy in new codes and themes across successive interviews. Within each group, no new codes emerged after approximately six interviews; this pattern held across all three country contexts, supporting the sufficiency of the sample.

### 2.3. Data Analysis

Data analysis followed conventional content analysis. Transcripts were read repeatedly to achieve immersion, significant statements were extracted, and meaning units were formulated. These were systematically grouped into categories and clustered into themes reflecting the breadth and depth of participants’ experiences [20, 24]. Analysis was conducted manually to preserve meaning and ensure interpretive depth; NVivo supported data organization. Although the analysis began descriptively, certain themes—particularly professional self‐worth, identity reconstruction, and existential restoration—invited a deeper interpretive layer that emerged inductively from recurring patterns across narratives, not imposed a priori. This transition from descriptive coding to broader conceptual interpretation was documented in the audit trail and confirmed through team consensus, keeping interpretive claims grounded in the data and consistent with the depth supported by conventional content analysis [24].

Triangulation was not used due to the qualitative descriptive design; however, rigor was maintained through several strategies. An audit trail was kept to document analytic decisions and procedural steps. A subset of five transcripts was independently coded by two researchers (MS and AU), with discrepancies resolved through discussion to enhance coding reliability. Following this, all transcripts were coded individually, and final coding decisions were reached through team consensus. The team included the principal investigator (AA), a senior nurse educator and qualitative researcher, an immigrant nurse faculty member (MS), and a PhD student (AU). Researcher positionality was actively considered throughout. The PI (AK) is a Turkish national nursing academic; her cultural familiarity with the Turkish nursing system likely strengthened rapport with participants, while also requiring conscious bracketing of assumptions about professional norms and expectations. MS is an immigrant nurse faculty member in the United States; her lived migration experience likely influenced her interpretation during analysis, particularly regarding themes of professional identity and self‐worth, necessitating ongoing reflexive monitoring. The team’s diversity, spanning migration and non‐migration experience, academic and clinical backgrounds, and multiple destination contexts, was leveraged as an analytic strength, enabling perspectives to be surfaced and refined. Reflexivity was integrated throughout the study, with the PI recording reflections after each interview to examine assumptions, emotional responses, and potential bias. Reflexive journal entries were used not only to monitor bias but also to document how migration‐related assumptions may have influenced interpretive choices, particularly for themes concerning identity reconstruction and professional self‐worth. Collectively, these strategies enhanced the credibility, dependability, and confirmability of the findings.

### 2.4. Ethical Considerations

Ethical approval was obtained from the Koc University Ethics Committee (IRB# 2024.330.IRB3.139). Online informed consent was obtained, and participants could refuse any question or withdraw at any time.

## 3. Results

The average age of the nurses (*n* = 23) was 33.48 ± 5.14 years, with an average of 2.73 ± 1.93 years since immigration. Among nurses, 82.6% were female, with 9.17 ± 5.37 years of nursing experience in Türkiye and the destination country (Table 1).

**TABLE 1:** Sociodemographic and Professional Background of Participants.

(*n* = 23)	*n* (%)	x̄ ± SS (*Min-Max*)
Age (years)		33.48 ± 5.14 (27–45)

*Gender*		
Female	19 (82.6)	
Male	4 (17.4)	

*Marital status*		
Married	16 (69.6)	
Single	7 (30.4)	

*Nursing degree*		
BSN	12 (52.2)	
MSN or higher	11 (47.8)	

*Time since graduation (years) (2004–2021)*		
2004–2009	4 (17.4)	
2010–2014	5 (21.7)	
2015–2021	14 (60.9)	

Total nursing experience (years)		9.17 ± 5.37 (3–23)

Nursing experience in Türkiye (years)		7.26 ± 4.97 (1.5–22.5)

Nursing experience in destination country (years)		1.71 ± 1.69 (0.00–8.5)

Time spent in destination country since migration (years)		2.73 ± 1.93 (1–9)

Five themes emerged from the interviews. Although some codes overlapped, the analysis emphasized key perceptions and reflections on how immigration shaped participants’ personal and professional lives. The qualitative content analysis, with emergent themes, categories, and codes, is presented in Supporting Table 3 (Supporting/Table 3).

### 3.1. Theme 1: Immigration Decision

This theme reflects how nurses navigated the interwoven pressures (push influences) and aspirations (pull influences) that shaped their decision to migrate. Push and pull factors influencing migration decision‐making are summarized in the Supporting Figure 1 (Supporting Figure 1). Reactions to their immigration decision were diverse: some experienced affirmation and support, while others described discouragement or subtle forms of resistance from family and colleagues.

Push factors from the home country included political instability and under‐resourced healthcare systems; professional concerns such as poor working conditions, low pay, limited career growth, lack of respect, and workplace violence; and personal concerns such as burnout, economic strain, and safety. Overall, feeling unseen and undervalued led to disillusionment, with a lack of respect, unsafe practices, and limited opportunities driving migration.

*National and Societal Challenges:* Nurses reported that Türkiye’s worsening economy reduced the value of salaries and purchasing power (*n* = 8), while political instability and demographic shifts driven by immigration (e.g., from Syria and Afghanistan) (*n* = 6) heightened their sense of insecurity.

*Personal and Family Well-Being Challenges:* Nurses (*n* = 5) primarily cited concerns about their family’s future and safety, which are linked to deteriorating economic conditions, declining purchasing power, and quality of life (QOL). Ongoing political tensions and the influx of immigrants are altering neighborhood dynamics, contributing to feelings of insecurity and uncertainty about raising children in that environment. Some (*n* = 2) felt trapped in repetitive routines, lacking personal growth and life satisfaction, while one (*n* = 1) described an emotional burden stemming from general systemic issues:

“*Honestly, the purchasing power, the economy… It’s become really tough lately… I thought I could build a future for myself, for my children who aren’t born yet, in a more liberal country, offering them a different kind of education and a future*.” (ID#6, The USA)

*Professional Challenges:* Seven nurses (*n* = 7) reported a lack of respect for nursing and felt that their expertise and contributions were undervalued within the healthcare system.

“*Nursing in Türkiye is, unfortunately, a profession that’s not really seen by patients and families… And sadly, management also doesn′t listen to nurses… The workload is really heavy, a lot of responsibility. The job descriptions are clear, but never followed…*” (ID#17, Germany)

For some (*n* = 3), career stagnation reinforced feelings of professional disempowerment. Advancement was perceived as unattainable, particularly in public hospitals influenced by politics: “*Political reasons play a huge role, especially in the public hospitals. No matter what you do, how good you are, there’s no chance of promotion. If you speak up even a little, you are pushed to another unit…*” (ID#16, Germany).

Others (*n* = 12) experienced role ambiguity, where an unclear scope of practice forced them into unsafe tasks, blurring professional boundaries, affecting their ability to work with their full potential: “*It’s something we’re not supposed to do, it’s very risky… legally speaking, especially if something goes wrong. For example, at night, we order and take blood; the doctors normally order which tests should be done, but we used to do it and decide to carry out blood transfusions…*” (ID#11, Germany).

The exhaustion from an overwhelming workload was a common experience (*n* = 15), often described as stressful and dehumanizing. It often resulted in work–life imbalance (*n* = 4) and poor personal well‐being (*n* = 19). One nurse recalled, *“We were always short-staffed… I worked over 100 h in a month, beyond the legal limit, and management just carried the extra hours forward so it wouldn’t look like a problem”* (ID#1, USA).

Another nurse described feeling *“trapped”* in a repetitive routine with little room for personal life: *“I felt stuck… barely seeing my family, even struggling to take my own leave. The hospital took up so much space that I couldn’t live my own life”* (ID#5, USA).

Economic strain deepened nurses’ struggles, with many reporting unfair pay (*n* = 13) and systemic favoritism that blocked promotion (*n* = 6). Despite advanced degrees and experience, they felt undervalued: *“I had a master’s degree and 2 years’ experience, but my salary was barely above minimum wage”* (ID#1, USA).

Pull factors to the destination country include national stability, stronger healthcare infrastructure, and clear immigration pathways; professional considerations such as higher pay, career advancement, societal respect for nurses, and positive work environments; and personal considerations such as better QOL and safety.

*National and Societal Considerations*: Migration decisions were shaped by a sense of belonging, safety, and cultural adaptation. Nurses compared healthcare systems and QOL across countries, with some prioritizing language proficiency (*n* = 4) and geographic closeness (*n* = 8). For some, learning a new language embodied embracing a new identity: *“If you go to a country, you go into its culture, language, you have to get used to everything. No one is begging you to come, after all”* (ID#10, USA).

Simplified visa and nursing registration procedures, along with a secure social system, made certain destinations more appealing. The anticipation of freedom, safety, and a better future for family carried profound emotional weight. One nurse reflected, *“There was a big shortage of nurses in Sweden… while preparing for all this country. Then we unexpectedly won a U.S. Green Card. It felt like life opened a door we hadn’t imagined”* (ID#1, USA).

*Familiarity and Social Considerations:* Familiarity provided comfort amidst uncertainty. Some nurses chose destinations they already knew through travel, study, or media (*n* = 6), while others followed family (*n* = 1) or sought out Turkish communities (*n* = 1).

*Professional Considerations:* Professional identity was central to migration decisions. Nurses longed for respect and recognition of their skills (*n* = 8), viewing dignity in practice as inseparable from self‐worth. Expanded career pathways (*n* = 6) offered hope for growth through specialization and advanced training. “*I never considered Europe… because conditions are worse than in Türkiye… not talking about salaries I’m a nurse who really loves the profession. I love bedside nursing… I enjoy succeeding in what I do.”* (ID#4, USA).

Nurses highlighted education and career development as pathways to flourish and to have their skills valued. As one participant shared, *“What brought me to the UK wasn’t just the financial, but the opportunities for education, personal growth, and career development. Otherwise, nursing salaries are pretty average everywhere*.*”* (ID#20, UK).

Some nurses sought fair pay (*n* = 3), manageable workloads, and supportive teams (*n* = 3). Together, these reflections illustrated a yearning for environments where work did not drain identity but nurtured it, restoring self‐respect.

### 3.2. Theme 2: Immigration Process

*Facilitators and Enablers for Migration:* For many nurses, the migration process was not only a formal procedure but also a deeply lived transition shaped by confidence, support, and reassurance. Education (*n* = 5) emerged as a source of empowerment, in which high‐quality training and language skills fostered confidence in credentialing. As one shared, *“I had no trouble during the process… our university handled everything smoothly”* (ID#6, USA).

Employer support (*n* = 3), such as credentialing assistance and sponsorship, eased transitions and was experienced as reassurance. Practical enablers, smooth migration processes (*n* = 2), and visible job opportunities (*n* = 2) further sustained confidence that migration was possible and sustainable. One nurse noted, *“My hospital worked with a company… they assigned a consultant who managed everything. I didn’t face any problems”* (ID#21, UK).

Peer and community support (*n* = 6) provided particularly valuable mentorship and shared strategies that made unfamiliar systems more navigable. Family encouragement (*n* = 1) reinforced emotional resilience, providing grounding for participants as they prepared to rebuild their lives abroad.

*Barriers and Hardships for Migration:* Migration was experienced not only as bureaucracy but also as a personal struggle marked by uncertainty and strain. The most common challenge was documentation delays and complex licensing (*n* = 8), which tested endurance and patience. One nurse reflected, *“The paperwork was really difficult… they even asked for work schedules from all the years I worked to recalculate my internship hours”* (ID#12, Germany).

Financial strain compounded these barriers, with nurses (*n* = 4) selling cars, relying on spouses, or depleting savings to meet relocation, exam, and visa costs. One recalled, *“We really struggled… my husband’s salary was the only one we had when I was on maternity leave. We sold our car for exam and visa fees”* (ID#23, UK).

Job searches and exam preparation posed another hardship, often with little guidance (*n* = 2). For many (*n* = 12), preparing for registration and language exams while still working in Türkiye was overwhelming. One nurse recalled the financial and emotional gamble of traveling to the U.S. for the NCLEX: *“The plane ticket was 10,000 TL… I worked 8 months to save, and thank God, I passed”* (ID#10, USA).

Overall, migration was experienced not only merely as a formal pathway but also as a profound vulnerability, in which financial sacrifice, emotional exhaustion, and uncertainty became central to nurses’ journeys.

### 3.3. Theme 3: Life after Immigration

*Adaptations to new life:* Adjusting to new cultures was experienced as both disorienting and transformative. Nurses described adapting to lifestyles, including eating (*n* = 4) and diverse accents (*n* = 9). For some, migration reinforced Turkish identity (*n* = 1), while others saw it as personal growth (*n* = 1). One noted, *“Not having supermarkets open on Sundays is something I still can’t get used to, even after two years”* (ID#17, Germany).

*Barriers and Hardships for Integration after Immigration:* Cultural and social adaptation was described as one of the hardest challenges (*n* = 15). One reflected, *“America is an individualistic society. You must do everything yourself, from painting your house to cleaning, food, everything. We thought we were prepared, but we encountered very different things, you’re all alone”* (ID#1, USA).

Language struggles (*n* = 13) deepened disconnection, while homesickness and limited social ties (*n* = 9) created heavy emotional burdens. One nurse reflected, *“I’ve been talking to the walls for a year… Loneliness is a big thing. It’s different. The biggest downside is living an isolated life. If something happens, no one cares”* (ID#19, UK).

High living costs, relocation expenses, and delayed employment created financial strain (*n* = 4). One nurse recalled, *“The first few months were really tough. I remember counting every bit of money I had… Now I’m not rich, but I no longer worry about making it to the end of the month”* (ID#3, USA).

Everyday life challenges (*n* = 7), such as navigating housing, banking, and bureaucratic systems, added to the overall stress of resettlement. Discrimination and bias were also encountered, adding to their struggles: One shared, *“Most of the time, if the applicant for a house is German, they’re preferred. Turks rarely get a response… Egyptian and Moroccan friends said the same thing. There is prejudice”* (ID#12, Germany).

While many valued the opportunities available for their children, some voiced unease about social risks such as adolescent drug use and shifting cultural norms. One parent reflected, *“The system here is built around children and education… but since middle school, I’ve seen drug problems, even in our own building”* (ID#22, UK).

*Facilitators for integration after immigration:* Inclusive cultural environments (*n* = 8) eased adjustment, with everyday courtesies symbolizing respect and belonging. One noted, *“At my son’s school, the first words they teach are ‘sorry,’ ‘thank you,’ and ‘please.’ That’s why people are so respectful here”* (ID#23, UK).

Improved lifestyle and well‐being (*n* = 7) also supported integration, where safe spaces and simple freedoms brought peace: *“Seeing green makes me feel at peace… the crowds, like in Istanbul, are very rare here, and being able to bike everywhere makes me extremely happy”* (ID#17, Germany).

Support from fellow immigrants (*n* = 5) became a crucial source of solidarity and understanding. One nurse shared, *“I have friends from Azerbaijan, Mexico, and Spain… since we’ve all gone through the same process, we understand each other. Our closeness to other immigrants is different from our closeness to Germans”* (ID#13, Germany).

Adjustment was not always smooth, with some describing it as deeply exhausting. One nurse reflected, *“In Türkiye, it’s like swimming in salty water… you struggle, but it lifts you a little. Here, I feel like I’m trying to swim in mud. That’s how exhausting and difficult it is”* (ID#6, USA).

*Professional life after immigration:* Nurses felt that their expertise was more valued abroad (*n* = 13), though some in the United Kingdom and Germany noted frustrations with differing educational regulations. For many (*n* = 12), belonging was fostered through recognition and supportive teams. One explained, *“Since I came here, I’ve felt more at hom*… *Even when problems arise, they try to listen and find solutions. Yes, it’s a tough work environment, but I’m valuable. They care about me.”* (ID#17, Germany).

Some nurses found pay and roles aligned with their education (*n* = 5) and embraced greater autonomy (*n* = 5), while others encountered unfamiliar duties (*n* = 6). In Germany, especially, the broader scope of tasks, from cleaning to advanced clinical work, reshaped identity. One reflected, *“We work harder here. I had never dealt with defecation in Türkiye. Here, I clean a lot. I clean urine. I mop the floor. I do everything. Sometimes, we even do ultrasounds.*” (ID#19, The UK).

Autonomy was both empowering and daunting. While independence fulfilled long‐held desires, some felt unprepared for its weight, leading to self‐doubt (*n* = 4), fear of responsibility (*n* = 3), anxiety about license loss (*n* = 2), and judgment (*n* = 1). One nurse shared, *“In Türkiye, I wanted autonomy… but here you’re 100% in charge, and I was scared. I realized it was much easier when responsibility given to doctors”* (ID#18, UK).

Positive working conditions emerged in system supports (*n* = 7) such as technology, lift teams, and patient care technicians, which lightened workloads and enhanced care. Clear roles and responsibilities (*n* = 5) brought confidence but also revealed contrasts with Türkiye’s practice. One noted, *“As a Band 5 nurse, I cannot aspirate tissue or suture… there was no such restriction in Türkiye”* (ID#22, UK).

Adjustment also meant embracing teamwork. A collaborative, diverse workforce (*n* = 3) and lower stress settings (*n* = 3) fostered satisfaction, yet sharing responsibility challenged old habits: “*In Türkiye, the patient was mine alone. Here, ten different people enter and leave the room… it took months to accept*.” (ID#10, The USA).

Workload experiences varied: some appreciated lighter shifts (*n* = 3), fewer hours (*n* = 3), and flexibility (*n* = 2), while others, particularly in Germany, reported heavier loads (*n* = 4), limited support staff (*n* = 2), and early starts (*n* = 2). One described, *“Here, I start at 6 a.m., washing immobilized patients, then treatments, I serve breakfast and take patients for examinations, since no transfer staff exist.”* (ID#15, Germany).

For some, lighter workloads felt unsettling, raising fears of skill loss: “*I thought, ‘I’m going to forget what I know. Here, it’s a bit easier*… *even though I’m much younger, there are many senior nurses who ask me things, like medication or procedures… explaining this to them makes me feel a bit better.*” (ID#11, Germany).

A few nurses (*n* = 2) reported a sense of lost professional status, having been recognized as experienced nurse in Türkiye; they found their expertise questioned by those with less training in new healthcare systems. One reflected, *“My honor was hurt… I have a 4-year degree, a master’s, ICU certification, 7 years in the OR. Then here, they ask, ‘Have you ever measured blood sugar?’ and it’s a nursing assistant asking”* (ID#13, Germany).

Cultural nuances (*n* = 2), discrimination (*n* = 4), and lack of workplace socialization (*n* = 6) left some feeling isolated: *“Sometimes, managers can be difficult because of cultural differences. Some cultures are very direct in their communication, and sometimes you think, ‘Are they scolding me?’ but that’s just their way of speaking*…” (ID#20, The UK).

Structured training and orientation (*n* = 9) eased adaptation by clarifying roles and expectations, even if initially frustrating: “*There is a lot of training. They talked about bureaucratic processes, like the healthcare system and banking. I was really upset: ‘Why do I have to?’ I’ve been a nurse for 22 years. It affected my motivation. But they were intended to help with the adaptation*…” (ID#14, Germany).

Across destination countries, notable differences emerged in how professional life was experienced. In the United States, nurses most frequently reported greater professional autonomy and role expansion, though these were accompanied by heightened anxiety about responsibility and fear of error. In the United Kingdom, the structured band system provided clear role definitions and a sense of professional recognition, yet some nurses experienced frustration with scope‐of‐practice restrictions that differed markedly from their training in Türkiye. In Germany, nurses reported the broadest role expansion, encompassing activities of daily living, cleaning, and certain advanced procedures, alongside heavier workloads, more limited support staff, and, notably, the greatest tension between perceived deskilling and skill‐based identity. The feeling of having expertise undervalued or unrecognized was most prominently voiced by nurses in Germany, where educational systems and nursing roles differ most substantially from the Turkish context. These country‐level contrasts underscore that integration experiences are shaped not only by individual factors but also by the structural and regulatory environment of the destination healthcare system.

### 3.4. Theme 4: Reflections and Future Outlook

*General Thoughts on Immigration*: For some, nursing enabled migration (*n* = 3), though leaving Türkiye caused heartache (*n* = 7). A few wished they had left earlier (*n* = 2). One participant reflected: *“Unfortunately, being this satisfied here is a bit heartbreaking, honestly. I wish I had come earlier before my mental and physical health got so worn down… This satisfaction is bittersweet*” (ID#18, The UK).

*Plans for Returning to the Home Country:* While a minority (*n* = 4) considered returning if conditions improved, most (*n* = 15) had no plans to return. “*I truly miss working in my native language. If one day Türkiye becomes the kind of place we hope for, I would definitely want to return to my homeland*.” (ID#12, Germany).

Professional and educational aspirations: Many aspired to pursue continuing education through graduate programs or other training (*n* = 14), and some (*n* = 4) sought to give back by sharing knowledge with Türkiye.

### 3.5. Theme 5: Recommendations for Destination and Home Country

Figure 2 (Supporting) highlights key priority areas requiring targeted action to improve nurse retention across both home and destination countries.

#### 3.5.1. For Home Country

*Nursing education and professional recognition:* Nurses called for stronger nursing education in Türkiye, including improved language training (*n* = 1), BSN‐only programs (*n* = 1), and preparation for international documentation (*n* = 1). They also called for greater societal respect (*n* = 3) through community service (*n* = 1), stronger regulatory and accreditation bodies (*n* = 2), and a national registration system (*n* = 3).

“*We need a nursing board in Türkiye. That board should properly track everyone’s education. People in Türkiye need motivation to learn. That’s the issue back home: people don’t want to learn, and don’t want to change anything*.” (ID#10, The USA)

*Workplace and professional environment*: Nurses emphasized that professional recognition requires more than titles; it demands systems that respect nurses’ education and expertise. Clearer role definitions (*n* = 4), fair ranking aligned with education and experience (*n* = 4), and roles that reflect qualifications (*n* = 2) were called for. Beyond structure, they suggest better working conditions (*n* = 3), accountability (*n* = 3), collaboration with doctors (*n* = 4), and nursing schools (*n* = 1).

Toxic workplace cultures (*n* = 2), silenced voices (*n* = 1), and biased management (*n* = 2) that many feel need to be changed, while supportive environments (*n* = 1) were described as essential to professional respect.

Economic and social well‐being: Nurses called for improved economic conditions (*n* = 4), and attention to burnout and mental health among nurses (*n* = 2) as essential steps toward improving retention and restoring professional respect.

“*Nurses are being paid around minimum wage, or slightly above. Administrators like, ‘Even if we only pay minimum, there’ll still be nurses waiting for the job.*.” (ID#9, The USA)

*Policy and advocacy*: There was a call for greater political attention to nurses’ concerns (*n* = 3) and for a shift in policy at the ministry level. Nurses highlighted the importance of strong advocacy through political involvement (*n* = 1), system‐wide reforms that engage all healthcare professionals (*n* = 2), measures to prevent workplace violence (*n* = 1), and the empowerment of national nursing organizations (*n* = 2).

Country‐specific thoughts: A few participants expressed concern about immigration to Türkiye, citing its impact on social and economic conditions, and urged the development of effective solutions.

#### 3.5.2. For the Destination Country

Nursing education: Nurses (*n* = 3) recommended improvements in nursing education specifically in Germany, such as requiring a BSN for entry into the profession. One nurse also emphasized the value of an advanced education for specialization, contrasting it with the United Kingdom’s entry‐level specialized programs.

Workplace environment: Nurses in Germany proposed creating nurse assistant roles to support tasks such as activities of daily living (*n* = 1), and some urged addressing short staffing (*n* = 2).

Support for language improvement: A few immigrant nurses (*n* = 4) recommend hospital‐provided language training to enhance their communication skills.

Process for recognizing license and prior experience: A few suggested developing a clearer process to recognize prior experience (*n* = 1), with better licensing procedures and a deeper understanding of the cultural differences they bring to the healthcare system (*n* = 1).

Mentorship and orientation programs: Nurses suggest longer and tailored orientation (*n* = 6), more mentored opportunities to understand the system, communication, and cultural nuances (*n* = 1), and financial support for further education (*n* = 1). One nurse noted orientation was treated like a simple ward transfer, not a shift to a new healthcare system, highlighting the need for structured learning: *“We’ve changed the whole country and the whole system. They assumed I knew everything, I reported it, and they started a program called ‘Teaching Fridays.’”* (ID#18, UK).

Support systems for well‐being: Broader support systems were seen as vital, including housing/banking help (*n* = 2), cultural adaptation (*n* = 1), and psychological services (*n* = 2). One UK nurse noted *“Language was tough at first, but the real issue is psychological; no one offers support”* (ID#19).

Policy and advocacy: Participants (*n* = 2) who immigrated to Germany recommended that nursing organizations be more visible and unite to represent nurses alongside other health professions.

## 4. Discussion

Türkiye’s nurse migration shows both global similarities and unique national factors, aligning with evidence of regional heterogeneity in migration drivers [18]. For Turkish nurses, national‐level dynamics such as economic instability, political uncertainty, demographic shifts, and reduced purchasing power fostered insecurity and intentions to migrate. Economic instability and low compensation were previously reported as key drivers of migration among Turkish nurses [25, 26]. These challenges mirror trends in countries experiencing economic recession and healthcare workforce losses [18, 27]. At the professional level, nurses reported feeling undervalued, underpaid, and constrained in their role identity and career development, consistent with research among migrant nurses from low‐income countries [15, 28]. Countries such as the Philippines, India, and several African countries have long been sources for immigrant nurses, driven by higher wages, better working conditions, and professional opportunities abroad [29]. Beyond the structural and organizational forces explained by existing theories [10] and reviews [14, 15, 28], Turkish nurses’ narratives showed that the decision to migrate was not merely economic or practical. It was deeply emotional and existential. Leaving became a way to reclaim dignity, safety, possibility, and a sense of self. Thus, migration emerged not from a single trigger but from the accumulation of lived experiences that reshaped how nurses understood their worth, their futures, and what they felt they deserved.

Nurses reported several pull factors shaping their destination choices that closely mirrored push dynamics. At the national level, stronger economies, supportive immigration systems, and, in some cases, geographic proximity and a shared language made a country more attractive. Shared cultural ties and common languages further eased mobility and linked to higher migration preferences [18]. Professionally, nurses sought recognition, career advancement, and nurturing environments. Evidence highlights that respect for nursing, supportive institutions, and growth opportunities can be as decisive as financial incentives [4, 5, 18, 28]. Consistent with Smith & Gillin [12], destination choices were shaped not primarily by economic rationality but by perceptions of place as a source of safety, stability, and a coherent sense of self, at times stronger than financial incentives. On a personal level, family–friendly policies and the promise of improved well‐being were key. These findings align with and extend de Haas’s aspirations‐capabilities framework (2021), which proposes that migration occurs at the intersection of aspirations, the desire for a better life, and capabilities that make acting on those aspirations possible. For Turkish nurses in this study, both dimensions were clearly present. Aspirations were not primarily financial; they were existential. Nurses aspired to practice in environments where their education was recognized and their professional worth affirmed. Capabilities were enabled by factors such as bilingualism, internationally recognized credentials, employer sponsorship, and peer networks that made migration actionable rather than merely imagined. However, the data suggest that the framework may require extension to fully capture what drove these Turkish nurses. De Haas conceptualizes aspirations largely in terms of a better material or social life; Turkish nurses’ narratives reveal a deeper layer. Migration was not simply a move toward better conditions but an act of reclaiming alignment between who they were as professionals and how they believed they deserved to be treated. This existential dimension, the professional self‐worth as a migration driver, remains underexplored in the literature and represents a meaningful area for further inquiry to refine current migration theories and frameworks.

Professional identity deterioration and restoration emerged as a central thread across narratives yet remain understudied in nurse migration research. Turkish nurses did not merely seek better pay or conditions; they sought environments in which their professional selves could be rebuilt. This aligns with evidence that professional identity is a significant driver of migration intent [17] and that restoration of autonomy, competence, and relatedness in destination countries is central to long‐term retention [5]. Australian evidence shows that divergent definitions of specialist nurse status, with internationally educated nurses prioritizing clinical experience and managers prioritizing formal credentials, contribute to underutilization and professional identity erosion [30], underscoring that this mismatch is structural rather than competence‐based. Future research should examine how professional identity deteriorates in home countries, how it is reconstructed in destination countries, and what organizational conditions best support that process, particularly across different healthcare systems.

Migrant nurses called for strengthening nursing in Türkiye not only through better pay and benefits to reduce economic insecurity but, more importantly, also through improved working conditions, professional autonomy, the requirement of BSN‐level qualifications, and enhanced respect for the profession. Similar themes emerge in studies involving nurses from Iran and sub‐Saharan Africa, where career advancement, fair compensation, and professional recognition are viewed as essential for retention [2, 31]. Migrant Turkish nurses also emphasized the need for stronger political engagement by nurse leaders and healthcare reforms that align with realities in clinical settings [26]. Sustained investment in workforce conditions and professional autonomy will be essential, and nurse leaders are uniquely positioned to drive this change by advocating for reforms that reflect clinical realities and retain nursing talent.

Migrant nurses recommended strategies to strengthen their integration and professional success abroad. Turkish nurses emphasized the need for a streamlined licensure that recognizes prior education and experience, as delays and devaluation were disempowering [5, 18]. Employer‐supported language training and bridging courses were viewed as essential for adaptation, consistent with evidence from the United Kingdom, Germany, and Australia [7, 32]. Mentorship and extended orientation were described as transformative, helping nurses navigate cultural and professional differences while fostering a sense of belonging and psychosocial well‐being [4, 13]. Emerging evidence highlights the value of digital transition programs in strengthening communication and professional skills among migrant nurses [33]. These integration challenges are not unique to Turkish nurses; they reflect systemic barriers faced by internationally qualified nurses in high‐income countries more broadly. Australian evidence consistently documents that even when nurses achieve organizational integration, many continue to experience under recognition of prior expertise, limited utilization of specialty skills, constrained career progression, and persistent challenges to professional identity and belonging [16, 30, 34], patterns that directly parallel the tension Turkish nurses described between gaining greater respect abroad and losing their sense of established expertise. While discrimination was rarely reported in this study, racial discrimination, constrained clinical roles, and lack of recognition remain documented barriers for migrant nurses in other contexts [13], underscoring the inequitable integration across borders. Structured orientation, recognition of prior learning, and equitable workforce policies are necessary to support meaningful professional integration [30, 34], and nurse leaders in destination countries are best positioned to champion equitable professional and long‐term career belonging for internationally educated nurses.

## 5. Conclusion

These findings suggest that migration among nurses is driven not only by economic or professional pressures but also by a deeper pursuit of dignity, safety, and self‐worth. Rather than a transactional decision, migration emerged as an emotional and identity‐based turning point, where leaving became a way to reclaim respect and possibility. This adds an important dimension to existing push–pull and macro–meso–micro models by showing that the desire for existential restoration is a central mechanism shaping the decision to migrate. Addressing it requires coordinated strategies across education, workforce policy, and organizational culture, with nurse leaders playing a central role in both source and destination countries.

For Türkiye, retention requires tackling unfair working conditions, excessive workloads, and a lack of respect for nurses. Priorities include expanding education and career pathways, opening leadership roles, ensuring competitive pay, and creating a national system to monitor migration for effective workforce planning. For destination countries, effective integration requires streamlined credentialing, structured onboarding, recognition of prior experience, and supportive workplace cultures. Equally important are measures to reduce isolation and stress through mental health and peer support, alongside strong antidiscrimination policies to promote equity and inclusion in the workplace. Strong nursing leadership and evidence‐informed workforce policies are essential to creating environments that retain nurses and support ethical, sustainable global mobility.

### 5.1. Limitations

Several limitations should be considered. The three‐country sample limits generalizability, and self‐reported data may be subject to recall bias. The relatively short mean time since immigration means findings primarily capture early integration experiences; longer established migrants may report differently. Volunteer sampling introduces potential self‐selection bias, as nurses with particularly strong positive or negative experiences may have been more motivated to participate.

Finally, researcher positionality may have influenced data interpretation. The PI (AK) is a Turkish national nursing academic without migration experience, while the team member (MS) is an immigrant nurse faculty in the United States; these positionalities were actively managed through reflexivity, audit trails, and team consensus coding.

## Funding

No funding was received for this manuscript.

## Conflicts of Interest

The authors declare no conflicts of interest.

## Supporting Information

Additional supporting information can be found online in the Supporting Information section.

## Supporting information


**Supporting Information** Supporting 1. Table S1. Comparison of nursing education systems, regulatory bodies, licensing requirements, language requirements, and career progression structures across the three destination countries examined in this study (United States, United Kingdom, and Germany). Supporting 2. Table S2. Sample semistructured interview questions used to elicit participants’ lived experiences and perspectives. Supporting 3. Figure S1. Push–pull model illustrating the structural pressures (push factors) and aspirational motivations (pull factors) that shape Turkish nurses’ migration decisions, organized into national/societal, personal/family, and professional dimensions. Supporting 4. Figure S2. Summary of participant recommendations for improving nurse retention and integration, organized by home country (Turkey) and destination country (United States, United Kingdom, and Germany). Supporting 5. Table S3. Full qualitative content analysis table presenting the five major themes, subthemes, categories, codes, and representative participant quotes from interviews with Turkish nurses who immigrated to the United States, United Kingdom, or Germany.

## Data Availability

The data that support the findings of this study are available on request from the corresponding author. The data are not publicly available due to privacy or ethical restrictions.
