# Supplementary material for: Lived Experiences of Nurse Migration to High‐Income Countries: A Qualitative Inquiry
Source: J Nurs Manag. 2026 Jun 25;2026:1582171. doi: 10.1155/jonm/1582171 (PMC13305131; doi:10.1155/jonm/1582171)
Supplement: Supplementary file 1 — Supporting Information Supporting 1. Table S1. Comparison of nursing education systems, regulatory bodies, licensing requirements, language requirements, and career progression structures across the three destination countries examined in this study (United States, United Kingdom, and Germany). Supporting 2. Table S2. Sample semistructured interview questions used to elicit participants’ lived experiences and perspectives. Supporting 3. Figure S1. Push–pull model illustrating the structural pressures (push factors) and aspirational motivations (pull factors) that shape Turkish nurses’ migration decisions, organized into national/societal, personal/family, and professional dimensions. Supporting 4. Figure S2. Summary of participant recommendations for improving nurse retention and integration, organized by home country (Turkey) and destination country (United States, United Kingdom, and Germany). Supporting 5. Table S3. Full qualitative content analysis table presenting the five major themes, subthemes, categories, codes, and representative participant quotes from interviews with Turkish nurses who immigrated to the United States, United Kingdom, or Germany. [file JONM-2026-1582171-s001.docx]

**Table 1. Comparison of Nursing Education and Practice Regulation Across Destination Countries**

| **Dimension** | **USA** | **UK** | **Germany** |
| --- | --- | --- | --- |
| **Entry-level degree** | ADN (2-year) or BSN (4-year); BSN increasingly required by hospitals and Magnet-designated facilities | BSc in Nursing (3-year); mandatory for NMC registration | 3-year vocational training (Ausbildung); BSc pathway available since 2020 reform but not yet nationally mandated |
| **Regulatory body** | State-based Boards of Nursing (50 states); no single national body | Single national body: Nursing and Midwifery Council (NMC) | State-based (Länder) recognition; no single national regulatory body |
| **Licensing/registration exam** | NCLEX-RN (national standardized exam) | NMC registration + Objective Structured Clinical Examination (OSCE) for internationally educated nurses | No national licensing exam; credential recognition varies by Länder |
| **Language requirement for IENs** | English proficiency (TOEFL/IELTS); CGFNS credential evaluation | IELTS or OET (minimum B2 level) | German proficiency (minimum B2 level) |
| **Career progression** | Clinical ladder system; specialty certification | NHS Band system (Band 5 entry; advancement through Bands 6–8) | Seniority and specialization-based; less structured than UK |
| ADN = Associate Degree in Nursing; BSN = Bachelor of Science in Nursing; IEN = Internationally Educated Nurse; NMC = Nursing and Midwifery Council; NCLEX-RN = National Council Licensure Examination; OSCE = Objective Structured Clinical Examination. | | | |

| **Table 2. Sample Questions Used to Prompt Participants** |
| --- |
| 1. Can you tell me about your journey to immigrate? 2. How do you describe your professional life while working as a nurse in Turkey? 3. Can you describe the process of making the decision to migrate? 4. How did you choose the country you migrated to? 5. How did your family and close circle react during the decision-making process? 6. Can you describe the challenges you experienced throughout your migration journey? How did these challenges affect you? 7. Can you tell me about your daily life after migration? 8. What challenges or supports have influenced your integration into society in your new country? 9. Can you tell me about your professional life after migration? 10. What challenges or supports have affected your integration into your new professional environment? 11. What do you think about the opportunities or challenges you may face as a nurse in your new country in the future? 12. What are your thoughts about staying in your new country versus returning to Turkey in the future? 13. What support would help you better adjust and thrive in your current country? 14. What could be improved in Turkey to better support nurses? |

| **Figure 1: Nurses’ Migration Experiences: From Structural Pressures to Aspirational Possibilities** | | |
| --- | --- | --- |
| Pressing Challenges (Push Influences) |  | Meaningful Aspirations (Pull Influences) |
| **National & Societal Challenges** |  | **National & Societal Considerations** |
| Living with financial strain and low purchasing power |  | Seeking stability in sociocultural and environmental contexts |
| Experiencing uncertainty from political instability and demographic change |  | Valuing language proficiency as a bridge to adaptation |
|  |  | Drawn toward locations that are nearer and easier to migrate to |
| **Personal & Family Challenges** |  | **Familiarity & Social Considerations** |
| Carrying worries for children’s future and family safety | **Nurses** | Leaning on familiarity with the destination country |
| Feeling stagnation and lack of life fulfillment |  | Following family/job opportunities that ease resettlement |
| Bearing emotional weight from systemic injustices |  | Finding comfort in the presence of a familiar community |
| **Professional Challenges** |  | **Professional Considerations** |
| Feeling undervalued and disrespected as a nurse |  | Yearning for recognition and respect as a nurse |
| Encountering blocked career growth and unclear scope of practice |  | Aspiring for expanded career opportunities |
| Experiencing exhaustion from overwhelming workload |  | Seeking fulfillment in fair compensation and improved work conditions |
| Struggling with unfair pay and systemic favoritism |  | Desiring meaningful work aligned with expertise |

| **Figure 2.** **Areas to Focus for Increased Nurse Retention** | | |
| --- | --- | --- |
| **HOME COUNTRY** |  | **DESTINATION COUNTRY** |
| **Nursing Education and Professional Recognition**  Improved language education  Improved education at universities (BSN only)  Improved respect and societal value for the nursing profession  Better prepared Universities for international documentations  Promoting community service among nurses for increased visibility  Regulatory bodies (e.g., Board of Nursing) and accreditation for schools  Registration requirement, such as NCLEX |  | **Nursing Education**  Better nursing education system |
|  |  | **Workplace Environment**  Creating nurse assistant or staff supporting nursing role  Solutions for short staffing |
|  |  | **Support for Language proficiency**  Language training supported by the employers |
| **Workplace and Professional Environment**  Strengthening role clarity (scope of practice)  Fair ranking systems based on experience and education  Ensuring nurses work in positions aligning with experience, and education  Improved working conditions (payment, workload, technology support, stress)  Strengthening accountability and control system  Enhancing doctor-nurse collaboration and relationship  Enhancing hospital - school collaboration and coordination  Change of workplace culture (toxicity)  Freedom of speech at work  Creating supportive environments for nurses to reach their full potential  Ensuring unbiased, competent management |  | **Process for Recognition of License and Prior Experience**  Better centralized system giving option to work anywhere in the country  System to acknowledge previous experience |
|  |  | **Mentorship and Orientation Programs**  Better orientation program for adaptation to professional life  Better understanding of cultural differences  Longer tailored orientation  Support for further education  More mentored guidance |
| **Economic and Psycho-Social Well-Being**  Improved economic conditions for nurses  Addressing burnout and mental health among nurses |  | **Support for Psycho-Social Well-Being**  General support (e.g., banking, renting house)  Cultural adaptation  Psychologic support |
| **Policy and Advocacy**  Greater political attention and responsiveness to nurses' concerns  Change of policy (view on) toward nurses at ministry level  Encouraging advocacy by nurses and nurse politicians  Focusing on system-level change including all healthcare professionals  Preventing workplace violence  Strengthening the role and voice of national nursing organizations |  | **Policy and Advocacy**  Nursing organizations to unite and support nurses |
| **Country Specific Thoughts**  Preventing the changing social cultural composition of country |  |  |

| **Table 3. Qualitative Content Analysis: Emergent Themes, Categories, and Codes** | | |
| --- | --- | --- |
| **Categories** | **Codes** | **Representative quotes** |
| **THEME 1: IMMIGRATION DECISION** | | |
| **Subtheme 1.1: Push factors from home country** | | |
| **National and Societal Challenges** | |  |
| Economic situation and Low Purchasing power (n=8) |  | "*This priority changed over time. When I first decided to go abroad, my priority was entirely focused on professional career development. That was my only focus. But later, as I kept working, I realized that money is also important—being able to live on that money. According to Maslow's hierarchy of needs, to comfortably meet all those stages of life, a salary is definitely not enough. Later on, money became more important. I'm being honest, really. Eventually, financial concerns outweighed everything else*." (ID#22, The U.K) |
| Political instability and changing population (n=6) |  | "*Migrant families, large families, had started to occupy apartment buildings. Well, we came here before seeing the most obvious part of that trend. But still… I had already predicted that kind of course of events —things like rising rent prices and so on happening in Istanbul. There were like 30–40 people living together. In the basement of our apartment building, there was that kind of environment. Anyway, because of all these reasons, we didn’t want to stay in Türkiye. And when we won the lottery, well, it was like butter on our bread. I had already submitted my degree equivalency applications—even if we hadn’t won the lottery*.” (ID#9, The USA). |
| **Personal and Family Well-Being Challenges** | | |
| Concern for future of family and children and safety (n=5) |  | "*In the last institution I worked at, I worked for 7 years as a nurse educator in a public hospital. Later on, as a result of my work—and without being anyone’s relative or having any connections—I started working there as a supervisor. In those roles, patients would come back to me and say, 'You taught me this, and after I started doing it, my life got better.' That’s when I felt emotional fulfillment. That’s when nursing gave me something in return. But… especially in recent times, you can’t get that financial fulfillment anymore. I had already completed 22 years… I could’ve worked another 5 or 10 years and then retired. But I don’t want my children to live like this in the future*." (ID#14, Germany) |
| Lack of personal growth and life satisfaction (n=2) |  | "*I felt a lot of pressure, you know? Like I was stuck in the same place. Life was just so routine. I wanted to grow, to do something for myself, but that just wasn’t happening. It felt like my whole life was going to be just going to the hospital and coming back*." (ID#5, The USA) |
| Emotional burden due to systemic issues (n=1) |  | "*I don’t even know how to say this politely. I just didn’t have the energy left to live in the country anymore. Whether it’s the mobbing at work or just daily life in our own country, all the stress and struggles... I mean, I felt like I needed to change that*." (ID#19, The UK)  "… *Will I be able to get a Schengen visa, for example? Whether I will get it or not is uncertain. Because I'm Turkish, that's the only reason. The only reason is that I am Turkish. They treat you like a refugee. When you apply for a visa, it’s really such a disturbing thing. That country wasn’t like this. It has become this way now. It really hurts. I mean, being exposed to it every day there. Every single day seeing something in the news destroys you. Even when you’re here, you feel sad. But there is a distance between you and that pressure. And for example, I really want to get the citizenship of this country. At least so that there’s a government behind me*." (ID#10, The USA) |
| **Professional Challenges** | |  |
| Lack of Respect for Nurses (n=7) |  | "*What affected me the most throughout this whole process was not being respected in Türkiye. I mean, I was working 24/7. Not a single soul would say, 'Good job' or 'Well done.' Not a single person would say, 'Well done, you’re working so well.' Of course, they don’t have to, but at least you don’t see it in your salary. That’s how it is, right? No, that’s not there either, nothing. I don’t know the exact Turkish equivalent of this phrase, but there’s something called 'Taking for granted.' Exactly. It’s as if people think you’re just supposed to do it. That really breaks a person. Like, why am I trying? Because I get treated the same as someone who’s not trying at all. So, the lack of respect I couldn’t get in Türkiye was the thing that really broke me. That’s why I had quit my job*." (ID#10, The USA) |
| Limited Career Development Opportunities (n=5) | Limited Promotion Opportunities (n=3) | "*To me, society has a big impact. The purchasing power has a big effect. And I think it’s much harder to progress in the profession in Türkiye*." (ID#3, The USA)  "*Of course, in terms of career, I didn't really see myself going very far as a nurse in Türkiye. The things you can do as a nurse are quite limited there. You will work in clinics, hospitals, or maybe something very limited. But when I researched here, I saw that nursing has no limits. You can work from home, you can work for companies, you can do research*." (ID#7, The USA) |
|  | Criticism and lack of support for graduate education (n=2) | "*For example, because I did my master’s, I was criticized, like, 'Why bother?' I worked with people who thought doing a master’s was unnecessary. They were my colleagues who said this, or, for example, when I tried to change things that were proven by research, or when I tried to do what I believed was right—when I tried to do high-quality work—I faced judgment, criticism, and received very different reactions during that time*." (ID#21, the UK)  "*While doing my master’s, no one helped me with adjusting my workday. For example, I remember having to attend classes completely sleep-deprived after a shift. I remember dozing off in class. I remember having to go to my next shift the very next day. These were really tough. Besides that, I also went through all the difficulties that all nurses face. But these are just the simplest examples I can give*."(ID#23, The UK) |
| Poor Scope of Practice (n= 12) | Limited Autonomy and Responsibility (n=4) | "*Charge nurses and team leaders have a say here. This is something that doesn’t exist in Türkiye. In Türkiye, there’s not even responsibility... Yes, there are positions. There’s responsibility, but no money. And then there’s no thanks either. You’re responsible for everything. The doctor also comes and yells at you. Yes, like you said, it’s worse than being beaten. In Türkiye, the higher your position, the more painful it gets. Nothing else changes. I mean, that’s how it is. Oh, and you have no say. You can’t improve anything. You can’t do anything. You just say 'okay' to everyone. Then why do nurses burn out in Türkiye? Well, in Türkiye, they burn out like this*." (ID#10, The USA) |
|  | Poor quality and support for nursing care (n=5) | "*We talk to patients, and here, they create something like that, constantly calming the patients. We don’t do anything to change the situation. We just give medication and wait for the patient to be quiet and sit in the corner. For example, we use restraints, we restrain them in Türkiye. There, these things don’t exist. Those were too much for me. And as I said, especially working with people who don’t know how to approach psychiatric patients, I think that was the biggest challenge*." (ID#20, The UK)  "*Overall, unfortunately, I didn’t have good experiences at work. Neither in private hospitals nor in the place where I worked. Maybe I was too young, maybe I focused too much on the negatives, I don’t know, but it never turned out the way I expected. Because I specifically wanted to do a master’s to work in psychiatry. And I did it, you know, to read something, to think that things could be done with different approaches. But the reality didn’t match with the education I received. That was really disappointing to me because I graduated from …. University. We were really focused on a holistic approach, like, 'Let’s do this, let’s do that.' We take communication courses for the first two years. But when we went to clinics, none of that applied. I mean, one nurse for 40 patients, and we were working 24-hour shifts*." (ID#20, The UK)  "*… many things are wrong in the care process. Let me give a very simple example. Let’s say the patient needs a bedpan. The patient's relatives are the ones helping. In my opinion, we should be the ones doing that because we need to assess the patient there. We even need to assess their urine. We need to assess their body. There are many things like that. This is just a very simple example. There were so many things like this for me. It just didn’t work for me there. Later, I became very unhappy there*." **(ID#5, The USA)**  "*For example, the tools were rusty. We had a solution called something. They’d dip the tools in the solution and take them out. They stay in it for about 5 minutes. Then, using sterile gloves, they take them from the solution and use them in procedures, and the same thing is used over and over again. Until it breaks: even if it’s rusty, it gets used again and again. Occasionally, the charge nurse would come by and say, 'These tools are rusty, send them for deep cleaning,' etc. But even then, they’d still be used a lot. Because we’d see it on camera. I mean, once you go inside, it magnifies—you’re doing laparoscopy. The tip is rusty. It’s just a sense of guilt, sadness. This is what I was talking about. When I was working in Türkiye, I worked with real sadness because there was nothing I could do. When I said, 'Let’s not use this,' no one took me seriously.*  *But here, for example, if I told someone in charge, 'This tool is rusty, a rusty thing is being used,' that tool would be immediately thrown out. A solution to the problem would be found right away. But in Türkiye, this doesn’t happen*." **(ID#22, The UK)** |
|  | Unclear expectations and role ambiguity (n=8) | "*When you become a bit more senior in a particular field and learn a few things, you start doing things outside of your job. For example, doctors often ask, ‘Can you do this?’ Go ahead—this is Türkiye. I mean, of course, these are things outside of our authority. But at the beginning, no one asks us to do them. They can’t, because we don’t know. We’re not familiar with it. But after a certain point, when we’ve learned some things, especially during night shifts, Actually, this isn’t something we should be doing. It’s something with a lot of risk. And when something goes wrong, we don’t know whether they’ll back us up. Of course not. So really, it’s something we shouldn’t do. We’re putting ourselves at risk there. Legally speaking, and when something goes wrong—it’s not our responsibility.*  *For example, in my department, at night we draw blood from all patients. Now, normally doctors would place the orders for which tests to do and so on. With those orders, we would print out the barcodes and draw the blood. Placing those orders isn’t normally our responsibility. But we still do it. then we decide, based on the patient's platelet or red blood cell count, whether a transfusion is needed—and we perform the transfusion ourselves…*" (ID#11, Germany).  "*Well, apart from that, nurse, doctor, job... It was the imbalance in workload. We were often forced to do many doctors’ tasks as well. I can say it’s due to an insufficient job description*." (ID#23, The UK) |
| Stressful and Overburdened Workload (n= 15) | Poor Workplace culture (mobbing, Toxic Environment, heavy workload) (n=7) | “*… I experienced different forms of mobbing in the private sector and different ones in the public sector. Each had different motives. For example, in the private sector it was more about “Clinging to power” — a kind of power competition. There’s rivalry. As soon as they notice you, they try to suppress you immediately, seeing you as a competitor. In the public sector, there was also a kind of mobbing — more like ‘don’t disrupt our routine.’ I have about 8 years of experience in the public sector, maybe more. There, we always tried to take our profession one step further — I can say based on the knowledge and guidance you (faculty) gave us. That’s clear. But while we tried to do this, we were met with: ‘No. Don’t disrupt our order.’ They are very rigid.”* (ID#16, Germany)  “*Honestly, being a nurse in Türkiye was very difficult. If I had to define it in one word, it would probably be 'hard' and 'stressful.' One reason was that I was working extra shifts. The working conditions were very intense. There was mobbing — both from management and from senior nurses. Another issue was the imbalance in workload between nurses and doctors. We were often forced to do doctors’ tasks too. I would say there was an insufficient job description. Also, working hours were inhumane, I would say. We had no time to rest. And economically, of course, it was not sufficient either*.” (ID#23, The UK) |
|  | Assignments Misaligned with Education or Experience (n=4) | "*Well, at the beginning, I started in a department I was completely unfamiliar with. I began working in the bone marrow transplant unit, and I had not received any training before. For example, this was a shortage specific to this department. I was aware of that when I started. It was also a different unit and caught my interest. I said to myself, I’ll start and learn. Somehow, I’ll improve myself in this area. But if I had received some training at the beginning—or had some prior experience in that area—it would have been better. Actually, I wanted to work in intensive care or emergency*." (ID#11, Germany) |
|  | Bad Management Practices (Top-Down Management Decisions) (n=6  ) | "*There was no such thing in Türkiye. You’re a nurse. It doesn’t matter what you want. What matters is what the management wants. Just keep going like that*." — (ID#17, Germany)  "*While working in Türkiye, the truth is, I think there is a form of implicit mobbing. Even though people don’t like to talk about it, there is mobbing from charge nurses, nursing service managers, supervisors, and even nurses who started 4 or 5 years before me. So yes, mobbing happens. That really stressed me out because I was a new nurse, still learning how to care for patients. Especially since I chose to specialize in a demanding unit like oncology. I actually really love caring for oncology patients."—* (ID#15, Germany)  "*The fact that people who are responsible, such as nursing service managers, have nothing to do with psychiatry. Not knowing how to approach situations when problems arise and the lack of a respectful environment. For example, because they are responsible, because they are service managers, because they are the head of the department, they may expect respectful language from others, but they themselves approach others in an informal manner. However, respect should be mutual."* (ID#20, The UK) |
|  | Long Working Hours and difficult shifts (n= 8) | "*The working hours were exhausting. 24 hours. I never worked that much because I worked at a university hospital for 5 years, so our maximum working schedule was 16 hours. And during this 16-hour shift, most of the time, I was working alone. Sometimes, between 16:00 and 24:00, there would be two nurses, but most of the time, I was working alone from 16:00 to 08:00, except for in intensive care. This was very tiring for me. The day after a shift, I couldn't even recover. The day after a shift, I was inevitably exhausted. The next day, I would again work from 16:00 to 08:00, and I didn't have much time to take care of myself. I was very unhappy about this*." (ID#12, Germany) |
|  | Short staffing (n= 2) | "*There is always a staff shortage in Türkiye. I don't know, how are state hospitals? I can only talk about my own hospital. Because there was always a staff shortage. And what happens is, we couldn't get paid for it. When we worked extra hours..."* (ID#11, Germany) |
|  | No support during COVID (n= 2) | "*During the COVID, there were management issues. The financial allocations were low. We already had 24-hour shifts before, but these became even more burdensome. We work on weekends, especially during those times. During the four days of the holidays, 24-hour shifts for two days. During the four days of the holiday, you can't spend time with your family.*." (ID#13, Germany) |
| Negative Impact of work on personal well-being (n=10) | Violence in Healthcare Settings (n=4) | "*In Türkiye, you do so much work, the only thing they don't do is to beat you. Actually, they even do that sometimes (referring violence in healthcare). That's why I said, I’m leaving, but I've learned a lot. I mean, here, as a nurse, I am someone who is respected a lot*." (ID#10, The USA)  "*There is incredible disrespect towards nurses and healthcare workers in Türkiye. There is an incredible amount of backlash. I still haven't been able to understand the reason for this. I think it stems from political issues. I mean, it might sound political, but if someone says 'let them leave if they want to,' then of course, society will put its backlash towards healthcare workers. I never thought or felt that I was respected in any way. The only respect I ever received was from my patients. But whenever I saw news of violence against a healthcare worker, I would watch it, crying*." (ID#21, The UK) |
|  | Burnout (n=2) | "*I’m not sure how to say this nicely, but I feel like I no longer have the energy to live in the country. Whether it’s the workplace mobbing or the daily struggles, struggles that exist in everyday life, I felt like something needed to change."* (ID#19, The UK) |
|  | Dissatisfaction (n=4) | "*If I were to describe the challenges I faced, first of all, the 24-hour shifts. I don’t think anyone should have to do that. And I won’t even talk about the low salaries. Given the economic difficulties in our country, it was generally unsatisfactory. And then, there were the incidents of violence, of course. There was little respect. Honestly, I didn’t think it was a profession that earned much respect. So I thought, at least I speak a foreign language. I should use it and go*." (ID#19, The UK)  "*We were very dissatisfied both socially and with the working conditions. In recent years, it became such that we had no choice but to be here now*." (ID#23, The UK) |
|  | Work-Life Imbalance (n=4) | "*In Türkiye, , I actually really liked it. I mean, when I first started, of course, I was inexperienced, but I learned a lot. It was probably also related to the unit I worked in. But of course, the difficulties of nursing in Türkiye, such as irregular schedules, forced overtime work, and working 24-hour shifts, are factors that quickly cause burnout*." (ID#8, The USA)  "*For me, the working hours were a big problem. I didn’t have time for my social life or for myself. That was a big factor. On top of that... I’m not sure how to say this, but the way people promoted at work, or how things were handled... For example, I worked there for about 4 to 4.5 years, and we were constantly sent to different units without our permission, or a newly appointed person being made head nurse and taking over your responsibilities. These were all demotivating things, things that didn’t make sense. We would get home, and they’d call us on the phone, telling us there was an issue. The issue had nothing to do with me. It was unprofessional, and these were the kind of things I experienced*." (ID#19, The UK) |
|  | Hardship to get off or holiday (n=2) | "*Do I need to do a night shift? Sure, I’ll do it. Extra hours? Fine, I’ll work them. I love my profession—no problem there. I don't mind taking care of patients, even if I have a lot of them. But for example, when it came to taking time off—I couldn’t. I needed to take leave, but I wasn’t allowed. I’m a human being too. I have a personal life. But it was like—‘No, you have to come in.’ At one point, I was a single mother living alone. I went through really tough times. And when it came to my rights, nobody cared. Because it’s seen as only* your *problem, and you’re expected to deal with it alone… You have a child, but you’re still at the work. And that’s one of the ugly sides of capitalism—it’s like you’re expected to only work. But no—I’m a human being. I have a life outside of work. Of course I’ll work, but in order to do that, I also need to be able to meet my basic human needs*." (ID#16, Germany) |
| Poor Pay relative to Education and Experience (n=13) |  | “Then*, of course, I started working in private sector. I saw that our workload was very high, even in the public sector, because they say it’s a lot of work too, but their salaries were almost twice as much as ours. Of course, they must get tired too. They are all my colleagues, and I don't discriminate at all. But there are places where their workload is high and places where it’s not. …. The huge gap in salaries is a separate issue. We’re doing the same work, maybe even more, because in the private sector, that’s usually how it goes. You end up working more, with a heavier workload. There are many things outside our main duties that we have to take care of.*  *Despite all my efforts, I wasn’t able to get anywhere, and I think that kept bothering me from all sides. I eventually thought to myself, "This isn’t working anymore." Whether it's work or anything else, we’re struggling in every way. Of course, financially, everything is getting more expensive, and we're seeing that firsthand*.” (ID#11, Germany)  “*When I look at Türkiye, the thing that motivated me the most to migrate was something my secretary friend said to me one day. She told me, “…. I saw your paycheck. You’re earning less than me." She’s a secretary, and we were working together*.” (ID#14, Germany) |
| Unfair System (Favoritism, Inequity) | A system driven by favoritism and personal connections (n=6) | “ …… *But someone who has connections, maybe a relative of someone in charge, gets handed a position. That’s exactly the kind of system that wears people down in Türkiye. While someone’s relative gets to take it easy, someone with no connections is left struggling, doing everything they possibly can for the patient, and at the end of the day, they go home with only the patient’s appreciation. I don’t think they feel seen or appreciated at all by their supervisors or managers*.” (ID#14, Germany) |
|  | Inability to find job (n=2) | *“I was very happy working as a nurse in Türkiye—at least until I finished my master’s degree. After that, I had to work at a private hospital. As you know, public job placements only happen every two years. I got in with the 2018 placement through KPSS (national exam for centralized job replacement for public institutions) and was hired by a university hospital directly through the university's own process.*  *But I couldn’t take the KPSS exams in 2020, 2022. I had health issues—I had a broken foot and was physically worn out. I skipped the 2024 exam by choice, but the earlier ones I missed because of my health. I think this is a big problem—having job placements only once every two years. It doesn’t make sense, especially when we keep hearing about how much nurses are needed. That’s why I had no choice but to start working at a private hospital last year. Private hospitals, well…” (*ID#1, The USA)  “*One of the things that really affected me was that they didn’t allow me to reunite with my spouse through spousal reassignment (to work in the same city). And even when I tried to make it happen, they put obstacles in my way. I can definitely say that—it had a big impact on me*.” (ID#23, The UK) |
| **Reactions to the immigration decision** | |  |
| Lack of Support from Family, friend and Employer (n=1) |  | “ *My family said that “the UK was waiting for you”. Honestly, no one believed it. To be honest, I didn’t believe in myself that much either. My girlfriend didn’t want to come. My friends were saying things like, “You’re just chasing empty dreams.” That’s how it was. No one wanted me to go, and they didn’t believe I could do it because, honestly, it’s really hard*. “ (ID#19, The UK)  “*Later, I talked to my supervisor and said, “This is the situation. I’m thinking of getting married and I’d like to take unpaid leave to study English.” Of course, they didn’t approve it. At first, they said, “I support you. Good for you, of course go and study. You’ll be a very good nurse,” and so on. But when I actually asked for the leave to study English, they turned on me and didn’t want to give permission. Then I wanted to be reassigned (transfer of workplace within the same system) to a community health center, and they tried to block that too. Somehow, I managed to transfer*.” (ID#22, The UK) |
| Mixed Support from Family and Friends (n=3) |  | “*My dad—my family—well, my dad was very supportive. My husband really wanted it too, just as much as I did. But my mom didn’t want it at all. Neither my mom nor my mother-in-law wanted us to go*.” (ID#22, The UK)  “ *I noticed people had really interesting reactions. Some cried a lot. Uh, I don’t know… I got some strange responses. Like, “Why are you leaving? Shouldn’t you be contributing to your own country instead of working in another one?” Or, “Ah, you’re going there to make more money. You’ll buy a house and a car in your first year… But honestly, I never had any financial concerns. From the very beginning, my only goal was to improve myself and learn new things. That’s what truly motivated me—and to do it all in a happy and fulfilling way*.” (ID#21, The UK) |
| Mocking or Negative Judgments (n=3) |  | “*And we decided to move abroad. I started studying English and told my operating room supervisor. I said, "Well, this is the situation—I want to move abroad." They already knew, because I was always studying English. Even back when I was trying for the U.S. But everyone looked at me like,* “Just keep working, nothing’s really going to happen.” *They thought I was just wasting time, fooling myself. No one at the hospital actually believed I could do it and leave.”* (ID#12, Germany) |
| Encouragement and Support Received (n=3) |  | “*Because working was still very overwhelming for me... At that time, with the support of my husband and my family—they really pushed me to do this. They said, “Take unpaid leave. There's no way you can keep working at this pace and also pass an exam like this. It would wear you out completely.” So, I took unpaid leave, and with their support….. During that time, my husband and my family were incredibly supportive. You know, the moms—they took turns coming over, cooking for us, filling up our freezer and everything, just so I could fully concentrate on studying*.” (ID#18, The UK) |
| **Subtheme 1.2: Pull factors to destination** **country** | | |
| **National and Societal Considerations** | |  |
| Evaluation of All Options (n= 1) |  | “*I originally started thinking I needed to go somewhere. English was the only language I knew, really. So first, I looked into Australia. I asked an agency, but they told me my nursing qualification wouldn’t be valid there. I was like, “Okay, then.” At the time, I had a girlfriend and we applied for a German course together. But then some friends who had gone to Germany started coming back. They weren’t happy there. I’m talking about colleagues from our hospital. So then people were like, “Germany’s not good either.” Then we considered Ireland. That didn’t work out. After that, I was really close to going to New Zealand. Like, seriously, I was going to go. Then I circled back to the UK—I had actually tried the UK first. Then New Zealand again. Then back to the UK. And that’s how I ended up here*. (ID#19, The UK) |
| Language Proficiency (English vs. Other Languages) (n=4) |  | “*What made me decide on the U.S. for sure was that it was actually my first experience abroad. And it’s a place where English is spoken—it’s the primary language there. I already knew English, so learning German, for example, didn’t make much sense. The UK could’ve been an option too, but like I said, I felt that cultural factors would make things easier for me in the U.S. So that’s why I chose it*.” (ID#2, The USA) |
| Geographic Proximity to Türkiye (n=8) |  | “ *We actually considered Canada at first—my husband was studying English... But for me, the distance was a big deal. Long flights are a bit tough for me. Also, my husband lives in Ankara, and I didn’t want our daughter to have issues seeing her grandfather. I wanted it to feel like we were just in another city—not so far away. …. Germany is close, and other European countries are nearby too. I think the standard of living is good here—of course, now there are some challenges due to immigration, but overall, the quality of life and food is still better, especially compared to the U.S. or some other countries. Places like Canada or Australia feel too far. Germany felt closer and more accessible, especially with visas. People here have learned how to bring workers over, so it’s easier to come here for us from other European countries*.” (ID#16, Germany) |
| Simple (Immigration) Process) | Professional guidance by employer or company (n= 3) | “*During that time, someone messaged me on LinkedIn. They said something like,* "I help bring nurses to Germany. If you’re interested in applying, feel free to reach out.” *That message stuck in my mind. So I got in touch with the person, and just two days later, they enrolled me in a German language course.*  *Honestly, the fact that this woman helped me start the process so quickly kind of made the decision for me—I ended up choosing Germany. Like I said, I did have a few other options, and of course, I was a bit nervous about learning the language. But since I actually enjoy learning new languages, I thought I could handle it. And with the confidence and support she gave me; it all just came together*.” (ID#17, Germany) |
|  | Availability of Permanent Residency (e.g., Green Card) (n=5) | *“Yeah, we won the Green Card lottery. It was actually my husband who got it. I was the one who applied—on behalf of both of us. Funny thing is, America was never somewhere I wanted to go. My husband didn’t want to either, and honestly, I still can’t say I really like it here. He’s always complaining, like,* “Why did we even come here?” *We definitely wanted to move abroad, but the U.S. wasn’t our first choice. Back when I was a student, I was really into the idea of going to Australia. That was kind of my dream*.” (ID#9, the USA) |
|  | Pre-existing Job Offers (n= 2) | *“I talked to a few more friends. One of my friends had also come to the UK—she was in London. I reached out to her to talk. And she was like,* “Why aren’t you considering the UK? Just send me your CV.” *After that, I got like four or five job offers from the UK*.” (ID# 18, The UK) |
| Socio cultural and environment | Quality of Life and better future for family (n= 10) | “*What really influenced my decision to migrate was the violence in healthcare happening in our country—not the financial aspect. I mean, if it were about money, we wouldn’t have considered Germany. But we want a safe environment. We want to have a child in the future, and we want our child to grow up in a safe place and get a good education..*.” (ID#12, Germany)  “*The professional challenges I’ve faced, and the issues with employee personal rights, played a big role. On a personal level, discovering new places and practicing our profession in a different language is also a great experience. I have children. Their future and education are big concerns. Right now, in Türkiye, one of the biggest problems driving migration is education. Schools are overcrowded, private schools are expensive, and it’s hard to keep up. And while trying to keep up, your quality of life suffers. There are so many factors, really... Things like the quality of food are better here—even the oxygen you breathe feels cleaner. It feels like you’ve climbed one rung higher on the ladder of capitalism. You know those movies? It’s like that. Quality of life is really important. Educational opportunities for children are the biggest reason. Professionally, we were not at ease either. We were not happy. There was no other way left*.” (ID#16, Germany)  “S*o, I got pregnant with my daughter. Then we said, especially with how bad things were going politically, "Let’s go." That’s kind of how it happened. But honestly, for years—even before I met my husband—I always wanted to come to America*…” (ID#4, The USA) |
|  | Cultural and Ethnic Diversity (n=3) | “*I didn’t think about other countries. Actually, I could have considered the UK because of its proximity to Türkiye. Or Germany, which was very popular for a while. I could have considered that too. But I think the USA, which is multinational states, provides me with a lot of advantages in many ways. That’s how I feel. As you know, here, there are people from India, Asian countries, Europe—people from all over. Therefore, my political views, religious beliefs, or anything like that wouldn’t hold me back. That’s why the USA has always been my first choice. But if that weren’t the case, I would definitely try the UK in second place*.” (ID#2, The USA)  “*The reason is also a bit about the lifestyle, the diversity, and the variety of people. No one feels like a foreigner here because, right now, you don’t have that luxury—everyone is already a foreigner. I’d say that’s one of the reasons of being in America*.” (ID#3, The USA) |
|  | Freedom and safety in social and professional life (n=1) | *“I’m gay. Being gay in Türkiye is probably one of the hardest things in the world. For years, you live a secret life. You hide everything inside. I’ve decided that I no longer want to do that. I decided that I want to be free, to be myself. And because of these reasons, I initially decided on Germany. I decided to move abroad, and one of the biggest reasons for that was this—to be able to be free, I wanted to leave the country immediately. Of course, culturally, I don’t feel very close to Turkish culture. Whether it’s family relations or the way people interact with each other, I always felt a bit different. I came here, and since I’ve been here, I feel more at home. That’s one of the biggest reasons*.” (ID#17, Germany) |
| **Familiarity and Social Considerations** | |  |
| Familiarity with the country (n= 6) |  | “*During our trip to New York, I fell in love with this country. And I saw that, culturally, we grew up with this country’s cartoons, its movies. So there’s been a certain cultural influence on us. That’s why, when we came here, I personally saw everything as resembling what I’d seen in those movies. I’ve always dreamed of living in this country. But realistically, it could’ve been elsewhere too—why not? It could’ve been the UK, Canada, or even Australia.”* (ID#6, The USA) |
| Husband having job offer (n=1) |  | *“I had actually decided to quit and stop working as a nurse. I had completely cut my ties with nursing. Since my English was good, I was working in health tourism. Later on, my spouse received a job offer from the UK. During that time... I did love nursing, but I wasn’t in a position to work as a nurse in Türkiye*.” (ID#20, The UK) |
| Having people already in the process for immigration (n=1) |  | “*It was a period when I wasn’t working but was continuing my PhD. One of our friends there shared with me that they were going to move to Germany. That’s when I started my process. I wanted to work there again. That friend was a research assistant working in Germany*.” (ID#20, The UK) |
| Presence of Turkish Community (n=1) |  | “*The biggest problem is the language, no matter what background I had, German is so difficult language. I had considered both the U.S. and Germany. But since the U.S. is very far away, in terms of my family being able to visit or me being able to go back quickly if needed, Germany weighed more heavily in my decision. Also, the Turkish population here is actually significant. Sometimes that can be an advantage, and sometimes a disadvantage, of course*.” (ID#13, Germany) |
| **Professional Considerations** | |  |
| Recognition and Respect for the nursing (n=8) | Respect for nurses (n=6) | “*I thought, “Let me go to an English-speaking country then.” For some reason, I didn’t research the UK much. I mean, I won’t say I regret it, but looking back, I think I probably would have chosen the UK if I had. And also, I ruled out Europe for another reason—nursing in Europe is viewed similarly to how it is in Türkiye. It’s not treated as a prestigious profession or anything like that… but because everyone gets a proper salary, it’s economically a bit better. Still, there’s not much of a difference. But like I said before, I’m also an idealist. I want to grow in the profession, to do different things in nursing.*..” (ID#10, The USA) |
|  | Established nursing education and system (n=2) | *“The fact that the nursing in the UK was well-established played a big role. Otherwise, we were going to choose between two other countries. The fact that it was the UK, it gave me a reason to return to nursing. Because honestly, I studied five years at …. University, did my master’s— it was starting to feel like all that was for nothing. I was absolutely determined never to work as a nurse again in Türkiye.*  *I think what stood out most was the potential for growth in nursing in the UK—because it actually has a future here. I came, I worked as a nurse, earned a salary. And it’s not just about the money. After some time, I could work with a specific patient group. Here, you start at Band 5 and can go all the way up to Band 8 or even 9. There are nursing roles across these bands. And as you progress, you become more specialized. Extra training is really important. These opportunities for training, development, and career growth matter a lot, not just financially*.” (ID#20, The UK) |
| Expanded Career Opportunities (n=6) |  | “*It was one of my dreams. I’m not sure whether I can actually achieve it, but for example, there’s something called CRNA here. You become a nurse anesthetist—basically like an anesthesiologist, except you can’t do a few specific things. Becoming one is one of my dreams, and as far as I know, if I manage to do it, I’ll be the only Turkish person to have achieved that… I even had this dream of returning to Türkiye one day to help advance nursing there and teach this role. But I’m not even sure I’ll be able to complete the first part of that dream—it’s a tough program. Still, it's that sense of excitement, the feeling of setting sail toward new horizons, that kind of thrill—that doesn’t exist in Europe*.” (ID#10, The USA) |
| Better Workplace Conditions and job satisfaction (n=3) |  | “*I guess it’s somewhat related to the sense of hopelessness people feel in their current situation. Because we know that the problems we’re experiencing in Türkiye aren’t things that can easily be fixed or are just temporary. That leads you to start looking for alternative solutions. That’s why I began to consider this path. Of course, I had no intention of changing my profession—I love my job. I was just looking for a place where I could do it better. In that sense, I compared all the countries—not just the U.S., but also the UK, Germany, and so on. I looked into where nursing could offer me better opportunities and where I could be happier. So we did some research at that time*.” (ID#8, The USA) |
| Compensation (Salary) Commensurate with Education and Experience (n=3) |  | “*And then, of course, salary was also a factor. Actually, not so much the salary itself, but the purchasing power. For me, the purchasing power is greater in the U.S. Also, the fact that they work 36-hour weeks was important*.” (ID#3, The USA) |
| **THEME 2: IMMIGRATION PROCESS** | | |
| **Facilitators and Enablers for Migration** | | |
| Educational Advantage (n=5) | Good nursing education and experience (n= 3) | “*They really appreciate how hardworking we are. They value our theoretical knowledge a lot. We’re preferred, actually. I think, as Turkish people—as Turkish nurses—we’re really recognized in that regard. The only real challenge, as I mentioned, is the system. The system is very different. The American healthcare system is incredibly different*.” (ID#1, The USA) |
|  | Good use of language (n=2) | “*I had actually decided to quit and stop working as a nurse.. Since my English was good, I was working in health tourism. Later on, my spouse received a job offer from the UK. During that time, I considered whether I could return to nursing... I did love nursing, but I wasn’t in a position to work as a nurse in Türkiye*.” (ID#2, The UK) |
| Efficient Fast Processes (n=2) |  | “*I think, in terms of procedures, the UK is the easiest country in the world. If I had known it would be this easy, maybe I would have done it much earlier. This kind of process doesn’t move as fast as it does in Türkiye, I think. At the beginning of the process, maybe because I accepted it and moved forward with that understanding, I didn’t face many issues. But let me tell you, my visa came out in 5 days, and after every exam, the next step was activated within 24 hours. I didn’t really have any major problems*.” (ID# 18, The UK) |
| Family Support (n=1) |  | “*I always thank my mom and dad. The best thing they did for me was to let me be free. I’m not sure if they fully realized what they were doing, but they always told me, for example, my brother and I remember him saying, “You’ll go, you’ll try. If you really want it, go ahead.” They always told me that—"If you really want it, you’ll go, try it, and if it doesn’t work out, you’ll always have a home here*." (ID#5, The USA) |
| Employer-Facilitated (Professional) Support (n=3) |  | “ *When we arrived here, they first picked us up from the airport and brought us to the hotel. They provided me with a 2-month accommodation, but not for my spouse. They wanted me to come without my spouse. I told them that I would cover all my spouse’s expenses and came here with him. We rented an apartment through Airbnb, just a room. I stayed in the hotel...”* (ID#22, The UK) |
| Peer and Community Support (n=6) |  | “..*The Telegram group created by our friend ..….. It's really a great support group for all of us. For all the Turkish nurses who want to work in the U.S., it provides guidance on what needs to be done, and it’s very diverse. Now, everyone’s situation is individual after a certain point. But up to a certain point, many things are similar*.” (ID#6, The USA) |
| Plenty of Job Opportunities (n=2) |  | “*You can apply to any hospital you want. To any position that you find fit, that you like, and where you want to work. You know the salary beforehand. You can talk about it in advance and even negotiate*.” (ID#7, The USA) |
| **Barriers and Hardships for Migration** | |  |
| Emotional Strain (n=3 ) |  | “*My mom came with me because she saw this country as a different life opportunity, and she really wanted to come. My mom, of course, struggled. And she still struggles. From that perspective, I struggle too. Apart from that, before coming here, I’d say these were the main challenges. Emotional struggles, like selling our house, selling our car, closing our home, and distributing our belongings*.” (ID#6, The USA)  “*For example, yes, we had a major issue with my eldest daughter's father regarding permission and the process. It took me about 2-3 years to get through that. We ended up in a legal process. Besides that, waiting for the visa was an incredibly long and painful process. The visa process was much longer in the past*.” (ID#16, Germany) |
| Financial Strain (n=4) |  | “*The exam fees were very expensive. I prepared for the OET and took the exam four times. It almost cost me a whole month’s salary. At that time, I was earning 11,000 lira, and I was spending 8,500 on the exam fees. I also took unpaid leave...”* (ID#22, The UK) |
| Documentation Challenges (n=8) | Plenty of paperwork and documentations need (n=5) | “*Since our documents, like the diploma and others from Türkiye, were different, the process of reviewing these documents took a very long time.. However, I was told that the system here is more accustomed to the documents from Filipino and Indian nurses, so they don’t experience as many problems with those, and that’s why their processes are faster. That’s the explanation I was given because they called me back for identity verification. There were discrepancies like different surnames and marriage certificates. But the main issue was that my diploma looked different. In Germany, there’s a process of diploma recognition, but here, there is no recognition process. They just require a notarized English translation of the diploma... It went through so many checks and steps, and that’s why it took a long time. But during that time, I continued working at the hospital. I was getting paid with a Band 4 salary*.” (ID#22, The UK) |
|  | Slow process (n=2) | “*In Türkiye, we're used to getting a document from the e-government system, instantly downloading it to our phone, and printing it out. There's nothing like that here. Sometimes you have to wait for 2 or even 3 weeks. I’m still not used to it. It’s really hard to adjust. You can end up waiting for an official document for a whole month*.” (ID#1, The USA) |
|  | Difficulties created by managers and previous employer (n= 2) | “*….., when I resigned from the hospital I worked at, my resignation wasn't accepted for a long time. I resigned and came directly. Since we hadn’t transitioned to permanent positions yet, some of my colleagues used unpaid leave, but I didn’t have that option. So, I resigned directly, and my resignation was not accepted. I also mentioned that I was going abroad. But my resignation still wasn't accepted. There were various issues like that*.” (ID#8, The USA)  “*I had colleagues who tried to block my path. Unfortunately, especially my charge nurse. I used to mention that I was attending a course. The course was on weekends, and I couldn’t be free every weekend, I was aware of that. But at least, if someone works on Saturday, they could write me for Friday’s shift, or a daytime shift on Friday, so I could be off on Saturday. On Sunday, they could put me on a night shift, so I could adjust my schedule according to the course timings. This way, I could attend the course, study better, and not fall behind at work. But my charge nurse insisted on scheduling me according to my course timings, meaning she scheduled me right during my course hours*.” (ID#12, Germany) |
| New Job search -Related Challenges | Unexpected change in prospective employer’s hiring (n=1) | “*So, I actually faced a really big difficulty. When I first came here, it was due to a huge bad luck. I had applied to ….. Hospital and got accepted for the job. But that week, due to a major setback, ….. Hospital decided to freeze their hiring process. They stopped all new recruitments for a long time, around six months. They even laid off some of their employees, and after that, they slowly started hiring again.*  *So, I found out that even though I had thought I was ready to start my job, it was no longer available. We had left everything behind to come here, so going back and waiting longer wasn’t really an option. I just decided to find any job to get by for the time being. This process lasted for about a month, and I started to feel really hopeless. Eventually, I lowered my expectations and ended up getting a job at …… Hospital through a night shift opportunity, thanks to a patient I was taking care of*.” (ID#9, The USA) |
|  | Being selective about the employer (n=1) | “*It was a really tough process. It was very difficult for me. The high exchange rate made things harder. And then, if I talk about England, England not get scattered. The bureaucracy is incredibly slow. The paperwork process is so slow. We send an email and have to wait two weeks just to get a simple response. From that perspective, it didn’t make things easier for me. Also, finding a job—this is something not everyone mentions—was incredibly difficult. Even though there was this campaign saying they were hiring a lot of nurses, we still couldn’t find a job. In comparison, finding a job in London was a bit easier. But the living conditions in London are a bit tough. Since I lived in Istanbul, I didn’t want to experience that lifestyle again*.” (ID#19, The UK) |
| No one to consult or ask (n= 2) |  | “*The people you're dealing with are more like official institutions rather than individuals, and that makes things harder. If it were handled more like consulting with someone, it might be a bit easier. But if a mistake is made, it comes back to you as wasted time. Unfortunately, these are very important factors. From that perspective, we did face difficulties. I was disrupting my routine, but thankfully, my spouse was there to support me. If I were alone or even with just one person, you'd still get lost in the process at some point*.” (ID#8, The USA) |
| Preparation for nursing registration and language exam (n=12) | Registration and other professional requirements (n= 8) | “*As someone who's experienced the ÖSYM system (national university entrance exam), speed really threw me off. For example, in ÖSYM, you have to be fast. You're racing against time. But here, it's the opposite. The institution that conducts the exam gives you 5 hours to complete it... I was used to trying to solve everything quickly to move on to the next questions. At first, I struggled with that. But once I understood the logic of the exam, I realized it was actually a passable exam that tests basic nursing skills. And I truly believe it's an exam all nurses should pass. Many people pass on their 3rd or 4th attempt. Those who pass on their first try are considered lucky. I was one of the lucky ones. I was really scared I wouldn't pass*...” (ID#1, The USA) |
|  | Language requirement (n=7) | “*I really struggled with learning English. The hardest thing for me was passing the English exam. To come here, there’s also the CBT exam. It's the nursing exam, written as CBT. I can honestly say I didn't really prepare for that exam. For example, I spent 2 or 3 days looking at a booklet, kind of like a driver's license test. There were questions in it. I checked them out. …… had an online course, and I joined it, but it was just a few hours long, not a huge course. I wouldn’t even say it was a nursing exam course. It was a very simple exam, and I passed.*” (ID#22, The UK) |
| **THEME 3: LIFE AFTER IMMIGRATION** | | |
| **Adaptations to new life** | |  |
| Adaptation to different cultures and accents (n=9) | Learning about different culture (n=7) | “*One other culturally nice thing I’ll mention is that in the market, everyone smiles at you. They say, “Good morning, how are you?” The other day, I was in line at a bank, just in casual clothes, nothing fancy, and a woman turned around and said, “You look so beautiful, you look amazing.” It really surprised me. It’s one of the nice cultural aspects here. I haven’t met a rude person yet*.” (ID#1, The USA) |
|  | Adapting to the different accent (n=3) | “*I think another challenge is the dialects; there are so many different dialects. That's why I find it so difficult. The things they say completely change. The words change, the way they say the words changes. For example, a patient might come from Bavaria, and it’s almost impossible to understand. Sometimes I find the language difficult because of this. That’s why Hochdeutsch (Standard German), the German that everyone speaks, is helpful. But then there are the dialects. Older people usually speak those dialects, and that can be a really tough process*.” (ID#17, Germany) |
| Developing new eating habits (n=4) |  | “*No matter how many Turkish markets you find .. you end up creating a Turkish American kitchen for yourself over time. I still make salad every morning. I’m from Antalya, so I eat the same thing every day—either my salad or eggs. Everyone says, "You're so healthy." But we actually eat like this at home, I don't make anything new. It's the same, I still follow the Mediterranean diet. I don’t eat bread. These are the same things, it’s part of our culture. But during the process, your sense of taste change. They change a lot*.” (ID#3, The USA) |
| More pronounced Turkish identity (n=1) |  | “*When I introduced myself, I realized that I also have a Turkish identity, and that has been a contribution to me. Being foreign and being Turkish, living as a foreigner—while everyone was talking about the European Championship in football, which I never follow and don’t know anything about, here…, if we won somewhere else, we were supposed to play with England. Our neighbors are very competitive, and we’re very competitive too. If that happens, we’ll set up in the garden and watch together. This added something unique to our lives. The experience of being foreign and also having our Turkish identity was a nice thing*. ” (ID# 18, The UK) |
| Personal Growth (n=1) |  | “*Learning to wait and be patient made things easier for me. It didn’t make anything else easier. I mean, we had to be patient. We had to wait. We learned to turn time into an advantage for ourselves. That’s how it got easier. Otherwise, there was no situation that would get easier. We had to wait*.” (ID#16, Germany ) |
| **Barriers and Hardships after immigration** | | |
| Cultural and Social Adaptation Challenges (n=15) | Adaptation to multicultural environment, social norms and lifestyle (n= 12) | “*When I first arrived, coming from a social, crowded city like Istanbul, it was really difficult for me here. Because, as I said, there’s no café culture. Okay, there are some Starbucks, restaurants, things like that. Actually, everything is available, but we don’t have friends here. So, I can’t just say, “Hey, let me go here with a friend.” The people at work? Our school is really big*.” (ID#6, The USA)  “*The cultures are very different. At work, for example, in our culture, we’re very open and friendly, but here, people don’t really talk about their personal lives at work. That was surprising for me at first. It’s actually a nice thing, though, because people keep things more private, within their own boundaries. You get used to talking and spending time with your coworkers, but for me, being in the same environment with them was very different. Their perspective on things, even the food, is completely different. For example, they heat up and eat fish, which is still very difficult for me.*  *Their behavior in public is also different from ours. I found it a bit odd at first. For instance, on the bus, they can talk loudly on the phone without headphones. Eventually, you get used to it. I realized that we, as people from our culture, tend to live thinking about others socially, while here, people are more relaxed about it. Maybe there needs to be some middle ground, I don’t know, but people talk loudly on the bus, watch TV shows loudly, and eat anywhere—on the bus, at a table, there’s no special place for eating. That was challenging culturally. Even with the people I take care of, their cultures are different too*.” (ID#20, The UK) |
|  |  | “*Sometimes, people can look at you for a long time, staring right into your eyes. But there's no particular reason for it, they just stare. It's funny because Germans are known for staring at people. It still feels weird to me—there's no reason, they just keep looking. And the more you look, the more they continue staring*.” (ID#17, Germany) |
|  | Challenges in creating a new social circle, making friends (n=8) | “*It’s like we’re not really friends outside of here. That could be one reason. Or it could be something like this: "I don’t have time, let’s go in a month." I’ve actually experienced this once. It happened to me. I asked, “If you’re free, how about we grab a coffee? We could practice German too.” This was someone involved in my orientation process, a nurse. She told me, “I’ll be free in two months, on these dates, if you want to go.” I was like, "Okay, we’ll see in two months*.” (ID#15, Germany)  “*It's the same with my home and neighborhood. I’m very lucky to have wonderful neighbors. They’re all from Manchester. But we're a bit shy, you know? We're hesitant to reach out because we don't know their culture, what’s appropriate or not, and what we should do exactly. That’s where our hesitation comes from..*” (ID# 18, The UK) |
|  | Creating Turkish community, Turkish culture (n=6) | “*Socially, the first few years were great. We spent a lot of time with all the Turks here and made very close friendships. But in the last few years, we’ve been more on our own, just with our family. We made many friendships, but unfortunately, we also experienced some sad things. Now, we mostly stay within our own circle, just us and our family. Sometimes, the relationships between Turks can be complicated. How can I put it? My wife and I, socially, feel a bit more distant. It's like in Türkiye too—sometimes we couldn't really protect ourselves in friendships. We were always being used. People took advantage of us. And since I'm someone who loves helping others, it made things harder*…” (ID#9, The USA) |
| Emotional and Psychological Struggles (n=9) | Being alone, far from family and friends (homesickness) (n=9) | *“ I came here alone. I don't have any relatives or anyone I know here. During this process, I made some friends. But I was the first Turkish person to come to this city, and I didn't have anyone to speak Turkish with. When I left the hospital, the first thing I would do was call my family so I could speak a bit of Turkish*.” (ID#11, Germany)  “*My eldest daughter returned to Türkiye this year. For her, adolescence has been a bit challenging, especially in terms of integration. We are a family that is truly passionate about Türkiye. We are very proud of our values, and we often compare everything to Türkiye, thinking it's better there. Our culture is much better. But when we look at the quality of life here, the schools are excellent. However, she ended up feeling a bit lonely. That was a major challenge. She actually learned German very well, and her school performance was great because the education here is much easier than back home, where it's very tough.*..” (ID#16, Germany)  “*I miss my mom, my parents a lot. I haven’t been able to return to Türkiye to visit my family yet. My sister had a baby, my grandmother passed away, and I couldn’t be there with them at those moments. These things are really sad. Things like that happen, but still*...” (ID#5, The USA) |
|  | Feeling totally alone (n=2) | “*I mean, when I came here, if you don’t have a spouse, family, or anyone you know with you, you are completely alone in this country*...” (ID#19, The UK)  *“I think this place is very lonely, both as a country and culturally. I really had to put in the effort to build my own circle of friends and social life. That part was challenging*.” (ID#2, The USA) |
|  | Hesitation with decision, thinking of going back (n=1) | “*I didn’t know what I would do if I went back. And I didn’t really want to go back either. I just felt like I had to keep going somehow*.” (ID#19, The UK) |
| Language and Communication Challenges (n=13) | Adaptation of use a new language and accent (n=13) | “*When I arrived here, I realized that what I had learned wasn’t actually the English they speak here—or that they don’t really speak the kind of English I expected. Experiencing that was quite something. Facing British English was really hard. There’s this pressure:* I’m going to speak, I’m going to manage, I’ll have a role to play—it’ll be like theater. *For me, doing that, performing that kind of role, was very difficult. It’s not something that suits me at all*.” (ID# 18, The UK)  “*We got a good English education. We didn’t study fully in English, but we had English classes. I really learned it well. I even took some courses. But if you don’t actually speak one-on-one, you can’t really learn the language. You just end up learning grammar. When I came here—especially maybe it’s like this in Florida—this place is very… multicultural. There are lots of Indian people, Filipino people… Everyone has their own accent. And I have my own accent too*.” (ID#4, The USA) |
|  | Self-doubt (n=1) | “*On my first day here, I felt terrible. You really feel like you’re all alone. And it was really tough. Then, I couldn’t understand or make myself understood—of course, I eventually learned. But even when I did, it didn’t satisfy me. The things I could understand or say just weren’t enough. I already know nursing… I’ve practiced it in Türkiye. Not being able to express the things I already knew—that really bothered me*.” (ID#11, Germany) |
| Financial difficulties (n=4) |  | “*After that, they had arranged a place for me to stay. But for example, I wanted to bring my dog—and that was a challenge for us as a family. You can’t just bring your dog directly. We had to pay £2,000, and the dog had to come in the cargo section. And financially, when we arrived… starting a home from scratch was really hard. You think you’ve saved enough, but when I came, £1 was about 45 Turkish lira—I think—and the difference was huge. No matter how well-off you think you are, that money disappears really fast.… that period becomes really stressful. We had saved, but honestly, it didn’t go very far. It runs out so quickly*.” (ID#20, The UK) |
| Daily life Challenges (n=7) | Banking, other necessities (n=2) | “*…., of course, the bureaucratic processes aren’t easy. When you come here, there’s always something—taxes, banking you have to handle, and other official procedures. Emails and correspondence… In Germany, everything runs on emails and letters*.” (ID#14, Germany) |
|  | Housing (n=3) | “*I had problems with housing because they don’t really want to rent to foreigners. For example, some places ask for six months’ rent up front, and some even ask for a full year in advance. So yeah, that was a big issue*.” (ID#22, The UK) |
|  | Unexpected challenges (n=3) | “*Culturally speaking, things like grocery stores and such—there’s nothing really within walking distance. As you know, especially in places like here ……., even just to go to the market you have to hop in a car and drive at least 10–15 minutes. That was something I wasn’t used to at all. Restaurants and cafés, for example—the café culture here is really different. In Türkiye, cafés would have games, and you’d go hang out with your friends for hours. Here, it’s not like that. As soon as you finish your tea or coffee, the waitress comes and drops the check or asks if you want anything else. It’s like they want you to eat and leave. That was something I really wasn’t used to*.” (ID#7, The USA)  “ *It even took us three weeks to get a phone line, can you believe that? Because we went in and said we want to get a phone line, but we didn’t have our Social Security number yet*.” (ID#6, The USA) |
|  | No work permit for partner (n=1) | *“One of the major things that’s really challenging for us right now is that my husband doesn’t have a work permit. They give us an H1B visa, and for the person dependent on us, they issue an H4 visa*.” (ID#6, The USA) |
|  | Subtle discrimination (n= ) ?? | *“I can't say I haven't experienced racism, but for example, no one has ever ignored me just because I'm Turkish. I don't really stand out much, being Turkish, blonde, and having colored eyes*.” (ID#1, The USA) |
| **Facilitators for integration after immigration** | | |
| Better Cultural & Social Construction (n=8) | Respect for personal boundaries (n=2) | “*But having my own personal space is important to me. These are all cultural differences because here, even if I go to a nightclub at night, I'm very sure no one will touch me or get too close, even though it's very crowded*.” (ID#2, The USA) |
|  | Diverse and multicultural life created by immigrants (n=5) | “*There are a lot of immigrants here, and it's a country with a large population. The patient population in my service is about 50% immigrants, and the nurses and staff are also mostly immigrants. Maybe that's an advantage. Here, we're all immigrants in a way. It feels like there's a common culture that has developed, with everyone trying to understand each other's culture. This perspective has evolved, and maybe that's why my work here has become a bit easier*.” (ID#18, The UK) |
|  | Welcoming culture (n=2) | “*I think society here is more educated. Compared to Türkiye, people are more educated, and that's why they're more tolerant of each other. They're also tolerant of foreigners. They've gotten used to it. In America, white Americans are used to immigrants. They think that immigrants must be very knowledgeable, especially if they’ve come from Türkiye, they must be experienced or intelligent*.” (ID#3, The USA) |
| Improved Lifestyle & Wellbeing (n=7) | Better social life, life and work life balance (n=7) | *“The peacefulness in the lush greenery here, it really calms my soul and heart. Before I came here, my health values were always off. Even when I went back to Türkiye for check-ups, my results were always bad. But now that I've started regular check-ups here, everything has improved on its own. My anxiety levels have dropped significantly*.” (ID# 18, The UK) |
|  | Feeling safe (n=1) | "*For example, when I walk on the street, I don't feel stressed or unsafe. But in Türkiye, in my own city, maybe even in my own street, I sometimes wonder, 'Is something going to happen to me now?”* (ID#11, Germany) |
| Social Support & Integration (n=5) | Social support from online groups, or other people immigrated before (n=3) | "*Nurses came together for a specific purpose are very supportive of each other. Our Telegram – online - group has over 2,000 members now. Not everyone is very active, but at least around 1,000 people are active. When there is such a focus, yes, everyone really supports each other*." (ID#9, The USA) |
|  | High Turkish population (n=1) | "*By luck, there are a lot of Turks around me, in the complex where I live. And there are also many Kazak people. They speak Turkish as well. So, it's like we're from the same background. In terms of human nature, I can relate to them*." (ID#1, The USA) |
|  | Immigrant community (n=1) | "*… social life is taking a bit of time right now. I have a Mexican and a Spanish friend. For example, you don't usually become close friends with doctors. In Türkiye, there is the nurse group, and the doctor group. Right now, I have friendships with three assistants. One is from Azerbaijan, another is from Mexico, and one is from Spain. It's different. Since we've all gone through the same process, we understand each other in a way. Our common language is German. We don't have a native language among us, but friendships are built like that. It's more about, how should I say, our closeness to other immigrants like us is different from our closeness to Germans*." (ID#13, Germany) |
| **Professional life after immigration** | |  |
| Appreciation for Education and Professional Background (n=13) | Quality education received (n=13) | "*The education we receive is truly very valuable. In all of our universities, we receive great education in nursing. And I have experienced it very clearly in this process. My nurse friends have also said the same thing. Nurses who come here from Türkiye have a very different level of knowledge. They are like doctors in Türkiye. They know everything. They can do everything. So, we have no issues in terms of education*." (ID#12, Germany) |
|  | Different education system (n=4) | "*Right now, there are actually a lot of differences in education. People is debating as well. Now, there is Ausbildung - a three-year program. They started with dual education. I think it's been about 5 to 10 years since this started. Dual education is considered both as Ausbildung and as a Bachelor's degree, but they are doing the same thing. For example, they are still trying to decide on how to make a distinction between them. They are trying to academicize nursing, they wanted to do that as well. But with Ausbildung in place, how can we do this? How can we separate these two groups? This is still one of the biggest issues. Should it continue as Ausbildung, or should we make it academic and continue as a Bachelor's degree? It keeps going back and forth. Bachelor's degrees can still be done. As I said, nursing here, but when I started working at the hospital, there is no difference in salary, and there is no difference in the job description based on education. These are the things being discussed right now. How can we change this*?" (ID#17, Germany) |
| Belonging, Recognition & Relationships (n=12) | Feeling heard as nurse (n=1) | "*Nurses are the boss of the hospital, doctor. There is incredible respect. No, for example, when the doctor arrives, nurses don't stand up for them. There is nothing like that. And it's the opposite. If a nurse says something, the doctor listens to it. Sometimes there are debates, but the final word is said by a nurse*." (ID#16, Germany)  "*We provide care, and it's really great that your voice is heard within the team. The decisions you make as a nurse are valued. For example, when you make an assessment, like a mental health evaluation or triage, your decision matters. If there's no issue with the patient's medication but they need speech therapy, for example, and there's no risk, they can be discharged and referred for therapy. The decision you make is accepted. No one challenges you saying 'this is wrong' or questioning how you made that decision*." (ID#20, The UK) |
|  | Feeling respected, valued, appreciated (n=11) | "*In Türkiye, even if you come from another hospital as a new nurse, you’re treated like a new nurse. Even if you have experience, you're still treated like a newcomer. But here, when I arrived, I felt like a fish out of water, with no idea what I was doing. Yet, they treat you with so much respect because of your experience. I remember thinking, 'Are you kidding me?' I’m saying I don’t know, so why are you treating me like this? It’s a cultural difference. For example, when you apply for a job here and say you have 10 years of experience, no one questions your 10 years of experience*." (ID#10, The USA) |
|  | Positive relationship with other HCP (n=4) | "*I actually find the communication within the team here to be very good. For example, no one comes to you and says, 'I’m going to do this for this patient, help me.' Let’s say a doctor needs to place a new catheter. They come to you and ask, 'When are you available? When can we do it?' It's something that is discussed and decided as a team before it’s done*." (ID#17, Germany) |
|  | Better management and positive relationship with managers (n=11) | “*Here, your supervisors—your managers—are technically responsible for your care and your scheduling, yes. But at the same time, they’re also very open to your feedback. If there’s something going on in your personal life and you need something, or if there’s a problem with a patient or something at the hospital, you can actually talk to them about it. There’s more of an open environment for discussion here*.” (ID#8, The USA)  *“In terms of working conditions, there's no mobbing here. At least, I haven’t come across any. My head nurse only comes into the room for two reasons: to ask if I need help or if she needs to step in for patient care. She always says, “How can I support you*?” (ID#11, Germany) |
| Tailored Training/ Orientations for Immigrant Nurses (n=9) | Orientations and Training and supportive environment (n=5) | "*I think our institution, hospital, and school here have done everything necessary for orientation. The team is the same way. Overall, about 90% of the time, people are helpful. Of course, not everyone is the same. When we don't know something, we feel bad. But as a country, Germany is used to foreign nurses and workers, and I noticed this—it’s very normal here. I definitely couldn't be as patient or understanding with someone who speaks a different language in my own country. But I guess they’re used to it here. They’re so used to us not knowing or understanding things, that they just show us again, explain things again, without getting annoyed. Our coordinator is the same..*." (ID#11, Germany) |
|  | Education opportunities (without judgement) (n=8) | "*There are so many incredible training opportunities and chances to improve yourself right now. For example, the place I work at is sending me to a training called 'Open Dialogue.' I’ve never heard of it before, and it’s something developed in Finland. They want us to apply it, but due to the number of patients, it might be difficult to implement. Still, the fact that they’re willing to spend money on it is really nice. Once completed, for example, you get a certificate in family therapy at the basic level. You have the chance to develop yourself. They constantly track training programs through the system."* (ID#20, The UK) |
|  | Mentorship and preceptors (n=2) | "*Nothing is like in Europe. Our entire systems (in Türkiye), Europe's systems, machines, materials, everything. So, my orientation was 12 weeks long, but I said, ‘How am I going to do this job?’ It’s very difficult here. But, for example, even this — having a 12-week orientation, even though there’s no such thing in Türkiye — it’s still different. Here, during orientation, there are binders. Each section has two or three preceptors assigned to you. You work with them during their shifts, and then they give you all the information you might need for your unit, listed in a binder. It’s like 30 or 40 pages. It includes things like medication preparation, medication calculations, pump programming, or things like setting up an IV, ventilator alarms, postpartum care, and so on. For each thing, as you do it, your preceptor signs off on it. So, even if you haven’t seen certain things, they explain them to you at the end of your orientation, like, ‘This is how it’s done*.’" (ID#10, The USA) |
| Pay and Position Commensurate with Education and Duties (n=5) | Better pay aligning with experience, education (n=5) | "*So, as bedside nurse here, I work 36 hours, 3 days a week. It's better, of course. They pay better financially. I mean, when I think about it, the money I earned as a manager was a joke. Now, I tell myself, if I were to go back to Türkiye, and they said, ‘We’ve hired you and put you in charge of the hospital,’ even if they offered me the same amount I earned before, they couldn’t pay me what I’m making now*." (ID#4, The USA) |
|  | Position aligning with experience, education (n=1) | "*Maybe the thing that motivates me more than Türkiye is this: I’ve been a nurse for 12 years, and still, being a master’s graduate and pursuing a PhD in Türkiye didn’t mean anything. But here, I started at Band 4 and moved to Band 5 within 3 months. Of course, that’s the expected process. I came here for a Band 5 position. And I can see now, there’s Band 6, then Band 7, Band 8... even if you don’t want it, there’s a career progression ahead of you, and you can see it. When I look at my colleagues here, with 8 or 9 years of experience, they’re at Band 7, Band 8, or in management and training roles, and they look so healthy, both mentally and physically. There’s no sign that they’re worn out*." (ID#18, The UK) |
| Scope of Practice & Professional Role | Different scope of practice (better or worse) (n= 7) | "*The difference here is that, depending on the hospital, the job descriptions can vary a lot. Some hospitals here have nurses whose job is to distribute food to patients and then collect the trays from the patients' rooms. In my hospital, that’s not part of the job because there are kitchen staff to handle that. Of course, if a patient is unable to eat by themselves and there’s no nurse assistant available during your shift, you help out. It then becomes part of your nursing duties.* (ID#17, Germany) |
|  | Unfamiliar scope of practice (n= 3) | "*I wish they had given us a training like what physiotherapists do, what social workers do, because I had no idea what they actually do. What situations do I need them for? Even when discharging a patient, we need approval from so many different people, from physiotherapy to social work. This process was really difficult for me. But the things I do love are definitely having my own patients. I’m responsible for them, I know them very well. As a nurse, I have more autonomy*." (ID#7, The USA) |
|  | Changing nature of nursing practice (n=3) | "*Actually, a bit because of our age, as students, we get really bored and overwhelmed with things we avoid doing. Here, for example, we have office days, and we create care plans for our patients. And the doctor, nurse, and the whole team have to provide care within the framework of the care plan you create. It really involves sitting down and going through that plan from beginning to end, including every detail, like a tomato allergy. It's a very thorough process. At first, it felt very different and hard to get used to. It felt like very computer (paper) -based nursing. I asked a friend who works in a general hospital here, and they said the same thing. I wondered if it had to do with the mental health aspect*." (ID#18, The UK) |
|  | More autonomy aligning with responsibility and accountability (n=4) | "*For example, here, the team leader nurse is me. I spent more than half of my nursing career in Türkiye as a responsible nurse or a team leader. I worked in the normal position for about two years, and then I moved to places where I was always put as the team leader because I became the most experienced there. Here, for example, the team leader has authority and a say in the matter. Even in routine things on the unit, charge nurses and team leaders have a say*." (ID#10, The USA)  "*… Here, when you make a mistake, you have to go to the family and tell them. I say, 'I gave you an overdose of medication. Then I realized it, and I'm telling you now. I gave you this medication at a dose that should have been different. I apologize. This could have certain side effects.' The doctor is aware, and the doctor comes to talk about it as well*." (ID#10, The USA) |
|  | Improved self-confidence and freedom at work (n= 3) | "*I don't think about going back. Because the working conditions here are much more comfortable compared to Türkiye, and I feel much more confident while working. I already have the knowledge and experience. I'm comfortable with that, but I also have the freedom to speak up and express myself here. For example, if my band 7 supervisor makes a mistake, I can easily point it out. And that band 7 will never, like, even glare at me. There's no such thing. I can speak up about it comfortably*." (ID#22, The UK) |
|  | More responsibility (n= 1) | "*This is a bit different from Türkiye in a way. It requires more responsibility, a higher level of responsibility. You really need to know exactly why something is being done. Sometimes, for new colleagues, this can become an overwhelming burden, suddenly taking on the responsibility of everything, knowing why something should or shouldn’t be done, and so on*." (ID#9, The USA) |
| Reactions to the new practice | Fear of the full autonomy (n=3) | "*At first, it was very scary. I told, 'Here you go, take it.' I used to say in Türkiye, 'I want autonomy, I want to manage patient care.' Here, you 100% manage everything. You make decisions for your patient, but I was so afraid. I was so used to being suppressed, having the responsibility given to the doctor. After years of wanting this, when they actually gave it to me, I was scared. I thought, 'What am I going to do now?' Actually, I realized it was much easier in Türkiye*." (ID#18, The UK) |
|  | Fear of losing license (n= 2) | "*Your license is your license... You can lose it. If you make a mistake, you can lose it. Because the rules are very strict. With just one mistake, there's a possibility of losing your license. So, as a nurse, you have to be very careful. In everything you do, especially with medications. You must check each medication twice. Check the order, how it should be done, is it this way or that way? If you're not sure, you must ask*." (ID#4, The USA) |
|  | Fear of being judged (n=1) | "*It seems like when there's a problem, we feel like we're going to be held responsible, like it's our fault, or that negative comments will be directed at us. It's the kind of psychology we get into, because that's how it is in Türkiye. Unfortunately, in Türkiye, if someone makes a mistake, rather than finding a solution, there's more of a tendency to criticize. In America, it's a bit different; they work more collaboratively. Instead of treating you like someone in charge, they approach it like a colleague, and they try to figure out how to solve the issue together, working with the management*." (ID#8, The USA) |
|  | Loss of social status due to being new nurse (n=2) | “*For example, when I first started at the hospital, I began as a Patient Care Technician. In Türkiye, I was working as a supervisor. When I came here, the head nurse told me, "You don't speak English," and for me, the biggest problem was the language barrier. I think that was the biggest shock. You come from a certain position in your own country, and when you arrive here, you're basically starting from zero. You start from below zero. In your own country, when you graduate and get a degree, you start with some level of expertise. But here, you come and start at a much lower level and don't even know the language. When you don’t know the language, people often treat you like you're not smart. Sometimes they don’t understand. But some people do get it, and they say, "You're really smart, it’s just the language issue*." (ID#5, The USA) |
|  | Self-doubt and frustration (n=4) | “*Because you can’t speak the language, people assume you don’t know anything, and they treat you disrespectfully, even with mobbing. Strange looks, behaviors, and when they’re talking to you, it’s all like that… I went through a lot of difficulties in the hospital. I cried a lot, faced many challenges, and had many arguments... but I’m doing better now. By around the 6th month, they started to understand. We had to go through this process. If I had the chance to go back, I think a lot of people, if they came here to experience a different culture, would go back if they had a chance. I think mobbing, in terms of Germany, would be a disadvantage. They can’t do that now*.” (ID#16, Germany)  “*No matter how confident you feel, when it comes to the language, there's always this situation where you doubt yourself unless, you're sure. You constantly feel the need to verify. I would always double-check, even three times, when I first started. Because you’re afraid of making mistakes*.” (ID#8, The USA) |
|  |  |  |
| Working Conditions | Diverse multicultural workforce (n=3) | “*Apart from that, most of my colleagues, other nurse friends, aren't even German. There are many nurses from Bulgaria, Morocco, and Arab countries. We all keep learning the language. The German colleagues who were born and raised here, or a few Turkish friends of mine who were also born and raised in Germany, help us a lot with the language. Even if I don't know the meaning of a word or don’t understand a sentence, they explain it to me in different ways. When I first started, my supervisor and the other colleagues told me the same thing: "We have to explain everything to you. If you don’t understand something, always ask so we can explain it in different ways." Even if I asked something 5 or 10 times, they never frowned at me. They never gave me a reaction like, "I told you this yesterday or half an hour ago, why don’t you get it?" I never faced that kind of reaction, and I never felt bad in that sense*.” (ID#12, Germany)  *“The American culture is very positive. Everyone is very polite, always asking “How are you?” and lifting each other up. But, it's interesting. For example, pain management might be handled by someone Chinese, neurology by someone Spanish, and orthopedics by an Indian doctor. There’s no real concept of a single "American" way — it's a melting pot. Everyone comes from different backgrounds, and it’s about how things progress and work out collectively. In Türkiye, since there aren’t as many diverse cultures, everyone generally shares the same culture*.” (ID#3, The USA) |
|  | System supporting nursing (n= 7) | “*In the U.S., there's something called a "Lift Team." For example, when you have an obese patient — and we do have a lot of obese patients here — it can be impossible to turn them or move them with just two people. So, a Lift Team comes in. These are big, strong people who are part of a special team. They help us when we need to turn the patient, assist with cleaning, or even with wound care. They’re there to support the nurses, making the patient's care easier and helping to improve the quality of care. It's a system that enhances patient care*.” (ID#4, The USA) |
|  | Clear roles and responsibilities (n=5) | “*In the U.S., everything about the working environment is much clearer, with roles and responsibilities clearly defined for everyone. It wasn't terrible in … hospitals in Türkiye, but in public institutions, I often hear complaints from friends where nurses end up doing everything — from secretarial work to handling medical records, and they’re expected to do things beyond nursing roles. There was even thing about nurses going to the pharmacy to pick up medications, questioning why they had to do it. In the U.S., these issues are much less common. Everyone has a specific role, and it's clearly defined, which is definitely a good thing*.” (ID#9, The USA) |
|  | Collaborative work with other health care providers (n=4) | *“In terms of workload, the nursing assistants here are not working on the same level as the nurses. Like in Türkiye, there are along with high school graduates, university graduates. Here there are LPNs (Licensed Practical Nurses), and nursing assistants. For example, with patients receiving insulin or infusion treatments, the LPN monitor blood sugar levels every hour, I monitor it every hour as well.. When I need to reposition a patient, they’re right there. The resources available here really impressed me*.” (ID#2, The USA) |
|  | Technology supporting care (n=5) | *“In Türkiye, the workload is very high. We fill out a lot of forms. I mean, we used to write everything by hand, like huge files, like a big book, that we’d write as nurses. But here, for example, the system is designed to make the nurse's job easier. There's a computer system, and everything is in the computer. Sometimes, the computer even understands that you might make a mistake. It gives you a warning, like "Are you sure?"* (ID#4, The USA)  *“These are different systems—very advanced. The technology here is much more developed. Everything runs through a computer system. I know things have slowly started to change in Türkiye too, but here, the system is well-established. I love everything about it. There’s nothing handwritten—like, writing by hand just isn’t a thing here.”* (ID#3, The USA) |
|  | No work after work (n=1) | “*The most important thing is that work stays at work. When we're done for the day, there's no work after that. I was honestly shocked when I realized there was no WhatsApp group. At first, I was like—what do you mean? Don’t you have a group chat for staff? How will they reach us? In Türkiye, even when I’m not working, my phone keeps pinging with messages. But here, if anything comes up, it just goes to your official system email*.” (ID#3, The USA) |
|  | Slow and stress-free work (n=1) | “*It’s like they have this general approach of making sure people aren’t stressed while working, and that they actually enjoy their job. That’s how I see it*.” (ID#23, The UK) |
|  | Fair promotion process (n=1) | “*It’s honestly really great. Actually, this week—I got a promotion! I started as a Band 5 nurse, and now I’m Band 6. To be honest, it didn’t feel that fast to me, even though some people get it in 6 months. For me, it took 10 months. I even thought, “Wow, 10 months feels long.” But everyone’s learning is different. Plus, I work in a different setting—I’m in the community. Being a nurse in the UK is really nice, and I work in a very unique place. It’s kind of like the CMHCs (Community Mental Health Centers) we have back in Türkiye*.” (ID#20, The UK) |
|  | Feeling safe (n=1) | “*Violence in healthcare is almost zero here. For me, that’s an incredible plus. Back when my husband was working in the emergency department in Türkiye, I was always on edge at home when he went to work—wondering if something bad might happen to him. He had some really serious issues while working in the emergency department*.” (ID#23, The UK) |
|  | Different population to serve (n=7) | “*Patients are incredibly polite. The most common things I hear from them are “thank you” and “please”—they're seriously very respectful. And there’s a clear boundary between nurses and doctors. The nurse’s job is clearly defined, and so is the doctor’s. If something isn’t my responsibility, I absolutely don’t do it—and no one can blame me for that*.” (ID#23, The UK)  “*Patient care in American culture is quite different. American patients often consider themselves very well-informed and tend to be more assertive or resistant. They might say things like, "I’m not taking this," or "I don’t want that," even if it’s been recommended by the doctor. And unlike in Türkiye, where you might insist a bit, here that kind of insistence can seriously backfire. The consequences of making a mistake or pushing too hard can be very serious and have significant professional outcomes*.” (ID#3, The USA) |
|  | Better relationship with patients (n=5) | “*But my relationships with my German patients, as well as those from other countries, are good. It’s actually very hard to get on my nerves. On the unit—well, you know, in the internal medicine department we get a lot of patients with dementia. I talk to them, and once we start talking, we usually understand each other*.” (ID#15, Germany)  “*In general, things are really different compared to Türkiye. For example, if someone has to wait three hours in the ER in Türkiye, they’d raise hell. But here, patients wait 7–8, even 10 hours in the emergency room and then get admitted to the ward—and they’re totally calm. They know the situation is bad, and they’ve accepted it. No one tries to attack or hit anyone. Overall—especially the elderly patients—they are incredibly polite and shy. Like, I don't know… sometimes a woman needs something, like she really needs to ask for it, but she doesn’t want to bother anyone. She can't bring herself to say it. It’s like that*.” (ID#19, The UK) |
|  | Serving Turkish patients (n=1) | “Turkish patients are actually a problem here too, by the way. My Turkish patients—yeah, they can be difficult, honestly. Sometimes I don’t even let them know I’m Turkish. I speak to them in German.” (ID#15, Germany) |
| Workload | Easy workload (n=3) | “*Even though it's outside my job, I’ve been doing things beyond that. Here, I started more focused on just care. Since there are a lot of geriatrics departments, the patients tend to be elderly. And sometimes it feels like I might forget what I know. Here, things are a bit easier. They don't expect much knowledge or experience from you. There are a lot of people who don’t know as much. For example, despite my youth, older people ask me things—like about medications or certain procedures. Explaining things to them makes me feel good, and I remember what I know. At least I realize I know something*.” (ID#11, Germany) |
|  | Options for Flexible schedule (n=2) | “*Right now, I’m working per diem. So, I can choose the days and hours I work. If I want, I can take a month off, or I can work one or two days a week or even do double shifts. Because of that, it's a bit like managing your own business, and you have more control over your rights here.*..” (ID#8, The USA) |
|  | Less working hours (n=3) | “*The relaxed work environment is amazing. It’s really a comfortable working environment. I work 8 hours a day, and for the night shift, it’s 9 and a half hours, so I still have time for myself*.” (ID#15, Germany) |
|  | Higher workload (n=4) | “*When I was living in Türkiye, working in hospitals, it felt like I was just sitting there. Because I used to have 4 or 5 patients, and when I had 5 patients, I would feel it was too much. But here, for example, on my last night shift, I had 18 patients. Half of these 18 patients were bed-bound, immobile, and unable to go to the bathroom alone*.” (ID#15, Germany) |
|  | Lack of nurse assistant or patient care personnel supporting care (n=2) | “*You've probably heard this already, but Germany has a very large elderly population. And since there's no concept like patient care assistants in hospitals, unlike in Türkiye.. Many people, when they come here, say things like, 'Is this my job? Did I come here to change diapers? I don't feel like I'm really doing nursing.' But I had already researched this before coming. Everything related to the patient is on the nurse’s shoulders. Inevitably, some patients can’t even get a glass of water from the table next to their bed, and for that, they might have to press the call button*.” (ID#12, Germany) |
|  | Early morning start at work (n= 2) | “*My tasks are very different compared to those in Türkiye. Here, I start my shift at 6 in the morning., I start washing my bed-bound, immobilized patients. Then, I do treatments. I handle all the medications immediately. By 8 o'clock, the patient's breakfast arrives, and I distribute the meals because patients don't have family or relatives here. After I serve breakfast, if the patient needs to go for an examination or radiography imaging, I take them because we don't have anyone in the hospital for transfers, and this duty falls to the nurse on the ward*.” (ID#15, Germany) |
| Challenges Faced as a Foreign-Educated Nurse | Use of language with coworkers and patients (n=7) | “*At first, of course, we had the language barrier. I still have, but they want to help, and we’re learning German. But coming here and actually living here is a whole different thing. When we first arrived, even my friends here, we felt like we didn't really know the language. We learned it, but it felt like we didn’t know it at all. It requires an even larger vocabulary. Because if I don't understand a word, they try to explain it to me with another word. That’s such a nice thing. And they say, "Give yourself some time," because I had asked them, "Why can’t I understand, why can’t I do it?" And they said, "You're doing great. You just need time. Don’t stress yourself too much." That really relaxed me*.” (ID#11, Germany)  “*There are many aspects in which I struggle culturally. The people we provide care for are often African,, or people we work with are from lower socioeconomic backgrounds. These are people I wouldn't normally encounter, because you don't meet them unless you live in that environment. It’s not like in Türkiye where someone might come to visit, or if you go on Erasmus, you’ll meet an English person. African cultures are very different from ours. As I mentioned, they speak very directly, and sometimes I don’t understand what they mean. I struggle with that. The English also speak in a very indirect way. It’s hard for me to understand things because they explain things slowly, and even understanding an accent is very challenging for me*.” (ID#20, The UK) |
|  | Fear of cultural misunderstanding (n=2) | “ *I was really scared about that. When I came here, I thought, "How will I understand patients? I can read their emotions from their tears, from a single word, I understand what they are going through, or what their complaints are in Türkiye." But I didn’t know the culture in the UK, and I didn’t speak the language either. I didn’t know if the things we have in our country would be there or not. There might still be things I don’t know. But I think one advantage I have is that it’s a country with a large immigrant population. In my ward, half of the patients are immigrants, and half of the staff are immigrants too. So maybe there’s an advantage in that, because we all share a similar immigrant experience, creating a common cultural bond*.” (ID# 18, The UK) |
|  | Discrimination (n=4) | “*You start from a very low position, and you don’t know the language. When you don’t know the language, people really think you're ignorant. Sometimes they can’t understand. Some people get it, they say, "Yes, you're very smart, it's just a language problem. I’m sure you’ll solve it soon." But some people don’t see it that way. They think you’ve come from somewhere else, that you don’t know anything, that you know nothing. Most of them don’t even know where Turkiye is. If you ask an American, of course, some people know, but they can’t even point to it on a map. They treat us like that. Once, someone asked me, "Do you speak Arabic*?"(ID#5, The USA)  “*…I became part of the team here. Of course, sometimes you face problems within the team. I wouldn't call it racism, but people sometimes approach you with prejudice. Unfortunately, this is one of the issues we've experienced*.” (ID#13, Germany). |
|  | Lack of socialization at work causing loneliness (n=6) | “*I mean, it's not like back home where you'd say, “Hey, let’s go out to eat,” or “Let’s all grab dinner together at a restaurant.” There isn’t even a cafeteria close to our building. We're a bit outside the main campus. There’s a dining hall further in, but even then, no one here ever says, “Let’s go to the dining hall together.” Everyone just stays in their own world. Sometimes I come in at eight and leave at five without even saying “good morning” to anyone. That kind of thing... yeah, it wears on you. It can really make you feel alone*.” (ID#6, The USA) |
| **THEME 4: REFLECTION ON IMMIGRATION AND FUTURE OUTLOOK** | | |
| **General Thoughts on Immigration** | |  |
| Nursing as the greatest opportunity to immigrate (n=3) |  | “*The main reason nurses in Türkiye leave is because we can use the advantage of practicing our profession internationally. Actually, we moved here from our country by using our profession due to the worsening economic conditions and declining quality of life. The main reason we fought and did this was for our children, to offer them a better future. As parents, that's our first responsibility*.” (ID#16, Germany) |
| Wishing they didn’t have to immigrate (heartbroken) (n=7) |  | Oh... migration is very difficult. Psychologically, and financially, emotionally. It's very tough. (ID#1, The USA)  “*Unfortunately, in terms of security, it's not just about hospitals. Even a woman walking alone on the street is a big problem these days. Here we go again, with the same issue, the penalties being not enough. If these things had changed, I wouldn't have wanted to leave Türkiye…. I would have preferred to work in my own language. Coming here, I understand this much better*.” (ID#12, Germany) |
| Wishing they had done it at a younger age or earlier (n=2) |  | “*Maybe it's my age, or if I were younger, it could have been easier. I've realized that I struggled a bit with learning. Not being able to grasp certain things, making things habits. A lot of the things I know, especially in terms of speaking, have been a challenge. I’ve felt the difficulties due to my age. Maybe if I were 5 or 6 years younger, it might have been easier*.” (ID# 18, The UK) |
| **Plans for returning to the home Country** | | |
| Depending on some factors (n=4) | Familial Pressure (n=1) | “*So, there is family pressure, the idea of returning in the future. Of course, my family is very supportive right now, but my mom keeps saying that she wants me to come back eventually. I am an only child, so that's why. Other than that, in my mind, I’m definitely staying here for the next 5 years*.” (ID#3, The USA) |
|  | Future Possibilities (including possibilities of other countries) (n=3) | "*Are we thinking about moving to another country? If we do, my husband's heart is a bit set on Australia or New Zealand. We've heard that the salary opportunities there are better from many people. But honestly, I don't know right now."* (ID#23, The UK) |
| No Intent to Return Home (n=15) |  | "*I had a dream one day, and it was so funny. Somehow, I had to go back to Türkiye and start working at my old job again. But I was crying so much, I woke up and thought, 'Oh my God, what is happening?' Anyway, it turned out to be just a dream. There were times when I worked sixty hours a week*." (ID#2, The USA)  "*I really don't want to return to the country right now. I only want to go back to see my family. In fact, if possible, I would like to bring my whole family here from time to time. The only thing I would want to do for the country is to improve nursing. Because I truly love nursing. If I can be part of something and make a difference, that's what I would like*." (ID#5, The USA) |
| **Professional & Educational Aspirations** | | |
| Pursuing Continuing Education (Graduate or Other) (n=14) |  | "*Maybe one day, in the distant future, I could continue with my PhD and move into academia. But right now, I don't feel ready for that. Because I really don't know the nursing here. There's a concept here called Nurse Practitioner. That's the goal I set for myself*." (ID#1, The USA)  "*As I mentioned, I'm taking family therapy training. If funding allows, I can develop myself further in this area. Right now, I've reached band 6, and after working at band 6 for a while, I honestly want a lead position. After that, I want to move to a smaller city. I don't want to live in London*." (ID#20, THE UK) |
| Exploring Ways to Support Nursing in Türkiye (n=4) | Plan for supporting nursing  (n=3) | "*I’m currently working in a hospice care company. It’s a different system, one that doesn’t exist in Türkiye... It’s end-of-life care. I want to bring this to Türkiye or at least talk about it and move forward in this field. I want to develop myself in this area. I definitely want to contribute to my Turkish colleagues*." (ID#2, THE USA) |
|  | Assisting Nurses with Immigration (n=1) | "*Nurse Camp is a company, and under this company, there's a subgroup called US Camp. It's a company that helps Turkish doctors, especially young doctors, whether they're recent graduates or students, with their processes of going to the USA. They've said that nursing is also growing a lot, and they want to add this to their services. They even offered me a position, and I said I’d be interested*." (ID#9, The USA) |
| **THEME 5: RECOMMENDATIONS FOR THE HOME AND DESTINATION COUNTRIES** | | |
| **Home country** |  |  |
| Nursing Education and Professional Recognition | Improved language education (n=1) | "… *I really think this is absolutely necessary. Outside of those who go into academia, no one feels the need for English education in nursing. There's no mindset like, 'Let me read a publication, let me read something,' because this is not common among other clinical nurses*." (ID#1, The USA) |
|  | Improved education at universities (BSN only) (n=1) | "*Or they're like, 'I've been a nurse for 30 years, I'm in charge of such and such,' but then they say they're only a high school graduate. And I say, 'Well, that doesn't work.' And they reply, 'How doesn't it work? But I have experience,' and so on. I mean, that's how the world works. Some things may have been done in a ridiculous way in Türkiye*." (ID#9, The USA) |
|  | Improved respect and societal value for the nursing profession (n=3) | "*In both academics and the ministry's perspective, and by society in general, I can only say it's respect. I think this is the thing that hurts people the most, especially nurses*." (ID#21, The UK) |
|  | Better prepared Universities for international documentations (n=1) | "*Because in Türkiye, there's a need to fill out forms or correspondence for equivalency procedures for America or other countries. For example, …. University is good in this regard. I email them, they fill it out and send it, it's very easy. But many universities in Türkiye, when they see that the form is in English, there are some who say, 'We don't fill out forms in English.' I said, 'How can that be? Don't you ever communicate with international institutions*?'" (ID#9, The USA) |
|  | Promoting community service among nurses for increased visibility  (n=1) | "*Maybe it (raising public awarenesses about nursing) would be reaching out to the public directly? or it could be through training or a seminar for people, for example. I remember when I was a child, a nurse would come to our house and speak with my mother. I remember thinking at that time ‘She was such a knowledgeable woman.' She was a nurse. And I think community health nurses are very important. That's why one-on-one contact is crucial*." (ID#4, The USA) |
|  | Regulatory bodies (e.g., Board of Nursing) and accreditation for nursing schools  (n=2) | "*First of all, I think all universities should be accredited by an accreditation body based in Europe or America. My university was accredited, and I'm saying this because I know the process*." (ID#1, The USA) |
|  | Registration requirement, such as NCLEX (n=3) | "*NCLEX... Yes, I definitely find it very useful. I think there should be something like this in Türkiye as well because people usually study when there is an exam. I've seen this in myself too*." (ID#7, The USA)  "*We've registered the diploma. Alright, it's done. But is this nurse working properly? Has there been any problem over the years? There is no institution following this*." (ID#9, The USA) |
|  |  |  |
| Workplace and Professional Environment | Strengthening role clarity (scope of practice) (n=4) | “ *When I look back, they were doing really great nursing back there. Even though there were shortages in terms of supplies or other things, they had a strong knowledge base and were practicing excellent nursing. When compared to here, the only difference is that our boundaries aren’t clearly defined. Since we work within such a broad scope, we experience a lot of burnout*.” (ID#6, The USA) |
|  | Fair ranking systems based on experience and education (n=4) | “*There’s no difference between private and public hospitals here. In Türkiye, that’s a huge difference. That could probably be changed — the private vs. public hospital disparity. Because in Türkiye, there’s no standardized salary scale. But here, they’ve standardized it. You have levels — RN First Grade, RN Second Grade, and so on. They’ve created a structure. I have no idea how that could be standardized in Türkiye..*.” (ID#1, The USA) |
|  | Ensuring nurses work in positions aligning with experience, and education (n=2) | “*So, regarding specialization—yes, here you truly specialize. I worked in Türkiye for 5 years. I was considering moving to Ankara, outside of Istanbul. But even if I did, I had no guarantee that I’d be able to continue working in psychiatry. For example, they would just place me wherever there’s a vacancy—maybe the ER, even though I have zero experience there. That doesn’t matter. They fill the gaps*.” (ID#18, The UK) |
|  | Improved working conditions (payment, workload, technology support, stress) (n=3) | “ *In Türkiye, working conditions absolutely need to be improved for us. Extra shifts — yes, the payment for extra shifts needs to be improved. Because these extra shifts are kind of forced. The nurse has to take on more so that the unit can keep running*.” (ID#23, The UK) |
|  | Strengthening accountability and control system (n=3) | “*Because healthcare is mostly government-controlled in Türkiye, quality control is quite weak. I mean, when a mistake happens, no one really follows up on it. Those errors often get covered up. And when that happens, it creates a more relaxed working environment—but in a negative way. People end up making more mistakes. Without proper oversight, unfortunately, this leads to consistently poor outcomes. … but honestly, I don’t think there’s a single solution. There are just too many layers that need fixing—starting perhaps with the leadership itself*.” (ID#8, The USA) |
|  | Enhancing doctor-nurse collaboration and relationship (n=4) | “ *Here, I actually find the communication within the healthcare team to be really good. For example, no one just comes up to you and says, “I’m going to do this for the patient—come help me.” Instead, let’s say a doctor needs to insert a new catheter. They’ll come over and ask, “When are you available? When can we do this?” It’s something that’s discussed among the team, and a decision is made together before proceeding. I’ve never seen anything like that in Türkiye. There, the doctor comes in and says, “I’m doing this now,” and that’s it. You just drop whatever you’re doing and follow them. Once the doctor leaves, you’re the one who’s left to clean up everything*.” (ID#17, Germany) |
|  | Enhancing hospital - school collaboration and coordination (n=1) | “ *If I were to compare nursing education in Germany to that in Türkiye, I think Türkiye's gap lies here. In Germany, they provide a 3-year training program for nurses, which I find quite insufficient. When they graduate, they have gaps in terms of knowledge and experience. However, their education is fully integrated with hospitals, meaning they actively engage with patients and gain hands-on experience through internships, which helps them build professional experience in the field.”* (ID#13, Germany) |
|  | Change of workplace culture (toxicity) (n=2) | “*Actually, there’s a lot to do, but first and foremost, I would suggest a change in our sociological structure. In academic settings or hospitals, for instance, I’ve worked in hospitals, and similar toxic work environments can be truly exhausting. But I don’t want to imply that life is perfect here or that work conditions are always great here—it’s a bit of luck. There are certainly places in Türkiye where such toxic environments don’t exist either*.” (ID#6, The USA) |
|  | Freedom of speech at work (n=1) | "*Listen to and pay attention to the concerns of clinical nurses. Honestly, I used to feel like I was afraid of some nurses I worked with. If I said something like this in Türkiye, I would lose my job. Someone else would take my place. I wish they could remove this feeling, maybe ease it a bit*." (ID#7, The USA) |
|  | Creating supportive environments for nurses to reach their full potential (n=1) | "*But like I said, with such a heavy workload and doing such a beautiful profession, being so oppressed and seen as so worthless makes a person think, 'Why am I even doing this*?'" (ID#11, Germany) |
|  | Ensuring unbiased, competent management (n=2) | "*Managers should be impartial, and they should really be able to listen in an unbiased way. And even if the nurse is wrong, if they express a concern, I should work on improving that. It's not about saying, 'Let's get rid of them and train the new one.' Because that new person will make the same mistake. It shouldn't be with the mentality of 'Let them go, who cares.' Every nurse here is valuable*." (ID#5, The USA) |
| Economic and Psychosocial Well-Being | Improved economic conditions for nurses (n=4) | "*Financial matters, but they can’t do this just for nurses. It needs to be something for all healthcare professionals in general. It should be something that should happen, and this isn’t something that can happen overnight*." (ID#3, The USA) |
|  | Addressing burnout and mental health among nurses (n=2) | "*There are so many people who think, 'Let the day pass, let me finish work, and go home.' I worked as a supervisor in Türkiye as well. I used to see this. After working all day long, struggling, and providing the best care to the patient, without any recognition or appreciation, I would see the nurses’ motivation drop over time. Whether you're a manager or a trainer, whether at the institution or at the university, people need to recognize the difference between those who do the work and support those who do, while encouraging those who don't. This awareness needs to be established in Türkiye. There is no work ethic in Türkiye*." (ID#14, Germany) |
| Policy and Advocacy | Greater political attention and responsiveness to nurses' concerns (n=3) | "*Of course, nurses' value needs to be increased. Managers need to listen to nurses. What are their needs? What problems do they have? How can we find solutions to these problems? It's not just the doctors' problems. Nurses have problems as well. And Türkiye needs to think about how we can solve these problems. It's not just about salary; it's the working conditions, working hours, mobbing, communication within the team. I believe all of these need to be improved*." (ID#17, Germany) |
|  | Change of policy (view on) toward nurses at ministry level (n= ) | "*For example, the attitude of the Ministry of Health, the attitude of the people in the country, and society—all of these, I think, affect nurses' motivation and work. Actually, everything, nurses' entire social life, both emotionally and socially, will be affected. Because when a person feels worthless, it impacts all areas of their life. That's why I think the Ministry should develop policies in this regard*." (ID#21, The UK) |
|  | Encouraging advocacy by nurses and nurse politicians (n=1) | "*What I've been advocating for years is that there has to be someone in the parliament who is truly our advocate. But really an advocate—someone who won't just sit there but will genuinely fight for our rights as nurses. Not just as healthcare workers because we're tired of being grouped as 'other healthcare workers.' We need a nursing advocate. Nursing laws, regulations, and personal rights need to be restructured, in my opinion*." (ID#1, The USA) |
|  | Focusing on system-level change including all healthcare professionals (n=2) | "*... And then, for example, going out into the field and really observing the nurses, the staff—because it's not just nurses there. It's the nurses, the support staff, the whole team involved in patient care. Identifying the points where they're struggling and finding solutions one by one. For example, in Türkiye, the pharmacists at the hospital I worked in didn’t really do their jobs properly. Now that I’m here, I can clearly see the difference. The radiology staff didn’t do their jobs properly. Neither did the lab staff. And everything fell on the nurse. Even the doctors didn’t really do their part properly*." (ID#5, The USA) |
|  | Preventing workplace violence (n=1) | "*Well, what could have been done to prevent violence in healthcare? How could people have been made more aware?... Honestly, I haven’t really thought deeply about it. But first and foremost, the public needs to be educated and made more aware in this regard*." (ID#12, Germany) |
|  | Strengthening the role and voice of national nursing organizations (n=2) | "*I think the Turkish Nurses Association needs to be stronger. But of course, they can’t just declare themselves powerful — I think they need to have that power from somewhere. Maybe… maybe they need to gain political strength? Perhaps the government or the Ministry of Health needs to grant them some authority or recognition. Somewhere along the line, I guess… first, the people at the top of the Ministry of Health need to acknowledge this. There needs to be someone from the nursing profession — someone who can express themselves, someone from nursing who can step in, take a seat at the table, or support them — basically, someone who can represent nurses*." (ID#4, The USA) |
| Country specific thoughts | Preventing the changing social cultural composition of country (n=1) | "*…. But right now, Türkiye doesn’t have that power. This is probably the only thing that could be done because one of the things that makes people most uncomfortable is the migration process, especially in Türkiye*." (ID#3, The USA) |
| **Destination** **country** | |  |
| Nursing Education | Better nursing education system (n=3) | "*For example, Germany is quite behind in this regard due to the lack of standardized education. Because in most countries, nursing education is a bachelor’s degree, but unfortunately, in Germany, it is still referred to as 'Ausbildung' – vocational training*." (ID#17, Germany) |
| Workplace Environment | Creating nurse assistant or staff supporting nursing role (n=1) | "*The operational logic of intensive care is the same, of course. But here, we don’t have many support staff. In Türkiye, we could never manage patient care on our own. Here, unfortunately, there are no patient care assistants. We are just nurses. Our colleagues handle it themselves. They do everything for the patient, including turning and repositioning them. Or when you can’t manage, you ask a colleague for help. One holds the patient while the other changes the sheets. I think the biggest issue here is the lack of support*." (ID#13, Germany) |
|  | Solutions for short staffing (n=2) | "*Of course, one of the difficult parts is the staff shortage. Due to the recruitment of staff from abroad, this causes a shortage of nurses. This sometimes makes working conditions more difficult. There are times when we work without taking breaks. Or there are days when we work under really tough conditions*." (ID#17, Germany) |
| Policy and Advocacy | Nursing organizations to unite and support nurses (n=2) | "*Who will defend our rights, our professional rights? Professional things have not developed. Professional organizations as well... they haven't developed and didn't feel the need to develop until now. Because these things usually develop as a result of a need, so I don't know, maybe this has not yet reached that point of needing development of an organization. Of course, nurses being more involved in professional organizations is also necessary*." (ID#16, Germany) |
| Process for recognition of license and prior experience | Better centralized system giving option to work anywhere in the country (n=1) | "*I think this is a bit of a problematic situation. It's a situation where, if you're applying in Hamburg, it's the beginning of a process. But let's say 3-4 months pass, and you want to apply in another state because the decision has changed. You have to start the process from scratch because maybe they want different documents or want the translation to be certified in another way. This is a quite frustrating process because it's really hard not to change your mind within a year once you've decided on a city. Also, the nursing equivalency courses, for example. I didn't have a problem at school, but I have friends who came here and after 3-4 months, their course could only start then*." (ID#17, Germany) |
|  | System to acknowledge previous experience (n=1) | "*Because we took life-saving things. I remember doing things like setting up and removing the dialysis machine by myself. Resident doctors, or assistant physicians. While they couldn't take arterial blood gas, they would ask us to do it. 'Can you, do it?' .. so we’re coming from an experienced background where we handle these things. But not being able to get credit for that here was really bad for me. Maybe a procedure could be developed for that*." (ID#1, The USA) |
| Support Systems for Psychosocial Well-Being | General support (e.g., banking, renting) (n=2) | "*The nurses who were in charge of us came, and they spoke with the bank employees on our behalf. They told them that we were the new employees here. They said that we needed to open accounts. They kind of pushed us on that. If they had left it to us, opening an account might not have been as easy, maybe because it was with the English bank here. But they came and solved the issue by speaking directly with the bank staff. We didn’t face any difficulty. The account was opened the very next day. Similarly, they could help with housing issues. They could explain to real estate agents that in our country the system is different, rent is paid monthly, and they could provide some guarantee to the agent regarding the salary here. Maybe they could assist with that*." (ID#22, The UK) |
|  | Cultural adaptation (n=1) | "*Before people arrive, there could be informational sessions about cultural differences for the staff. I think this is really important because the person coming will have a different culture. Both the arriving person and the staff here need to be aware of this. It would help make the integration process a little easier if the cultural differences are understood by both sides*." (ID#17, Germany) |
|  | Psychologic support (n=2) | "*The brain adapts and all that. I had a lot of trouble with the language in the first few months. But beyond that, there’s the psychological support. No one offers it here. To be honest, this country constantly takes immigrant nurses, so for us, it feels like something special, an individual event. But here, no one cares. For them, it’s just a normal situation*." (ID#19, The UK) |
| Support for language proficiency | Language training supported by the employers (n=4) | "*In Türkiye, what you learn in the exams contributes to passing the exam. But when you come here, it doesn’t really help with speaking the language fluently. Honestly, my hospital did offer this opportunity. When I arrived, in addition to the nursing equivalency course, I was attending a language course two days a week… Outside of that, I was working at the hospital. They combined everything, which was helpful, but I think not every institution does that. So, I believe that after arriving, support for a language course should definitely be available*." (ID#17, Germany) |
| Mentorship and orientation programs | Better orientation program for adaptation to professional life (n=6) | "*Yes, I think in the past there used to be something like an orientation period, like a year, where you would be treated more like a student before being assigned patients. I think at least something like that could be done for about 6 months. Because they directly take you as a registered nurse, give you work, but you know nursing, but you don't know the system. And that's very difficult because you’re working with the system. Everything is protected by laws*." (ID#20, The UK)  "*.. they see it like a department change. Yes, it's enough for a short time, but the country and the system change, and they might ignore that. I felt like I had to complain to a higher manager about this. It felt like, 'I don’t know anything, but everyone thinks I know everything.' After that, something different happened. They started a program, where every Friday, we sat with international nurses and talked about something. They created something like 'Teaching Fridays,' where we learned how things were done. I don't know how much it helped, but they did their orientation and planning. After 2-3 months, we had already started to understand and do some things*." (ID#18, The UK) |
|  | Better understanding of cultural differences (n=1) | “*They might need to know this better. We work at very different levels and with very different perspectives. The nursing we do is quite different. It would be good for them to be more aware of this—what it’s like, what’s involved, and what’s different. The orientation process was superficial. It seemed like they assumed we just follow a standard procedure here, without fully understanding the complexities of how we work. Of course, there was an orientation, but I don’t think it was enough. They should realize that when a nurse comes from Türkiye or India, they might not know what kind of work we’re doing or how we’re working*.” (ID# 18, The UK) |
|  | Longer tailored orientation (n=1) | “*Now, hospitals are taking new nurses into residency programs. Some institutions, especially for those coming from Türkiye, don’t consider their prior experience at all, or they might only value experience up to 1 or 2 years, maybe 3 years at most. Now, they offer this residency program. So, they provide a one-year orientation. It might be more costly for the institution, as it’s a year-long orientation, whereas it used to be just 2 or 3 months. I had an 8-week orientation initially. Maybe if I had done a longer one, it would have been more beneficial, and I wouldn’t have faced some of the issues I encountered*.” (ID#19, The UK) |
|  | Support for further education (n=1) | “*Yes, schools are very expensive. Some kind of support could be helpful because they need nurses as much as they need Nurse Practitioners, and there is also a high demand for those with doctoral degrees and researchers. But getting to that point requires budget planning. It's really expensive, but this is a general issue in the U.S. It's not just related to nursing, unfortunately… maybe there could be some kind of support.”* (ID#8, The USA) |
|  | More mentored guidance (n=1) | “*I need to have a mentor. A fixed mentor who will explain the process to me slowly. For example, how we do things in Germany, how we handle things like central venous catheter dressing. They should speak slowly and clearly, in a way that I can understand. I need someone to guide me through this. Unfortunately, they don't have enough staff to do this*.” (ID#16, Germany)  “*Honestly, I had imagined everything differently. I thought there would be someone, a Band Six nurse, as they call it, who would be with me all the time, showing me everything and being there for me when I did everything for the first time. It was like that in Finland when I did my internship. I was a student there. They probably do the same for new nurses here as well. For the interns, they also do this here. They kind of expect y*ou to know everything, just talk about the process for a day, and then let the intern work as part of the team.” (ID# 18, The UK) |
